# Association of Common Zoonotic Pathogens With Concentrated Animal Feeding Operations

**DOI:** 10.3389/fmicb.2021.810142

**Published:** 2022-01-10

**Authors:** Yaqiong Guo, Una Ryan, Yaoyu Feng, Lihua Xiao

**Affiliations:** ^1^Center for Emerging and Zoonotic Diseases, College of Veterinary Medicine, South China Agricultural University, Guangzhou, China; ^2^Vector- and Water-Borne Pathogen Research Group, Harry Butler Institute, Murdoch University, Murdoch, WA, Australia; ^3^Guangdong Laboratory for Lingnan Modern Agriculture, Guangzhou, China

**Keywords:** concentrated animal feeding operation (CAFO), zoonosis, emerging infection, public health, epidemiology

## Abstract

Animal farming has intensified significantly in recent decades, with the emergence of concentrated animal feeding operations (CAFOs) in industrialized nations. The congregation of susceptible animals in CAFOs can lead to heavy environmental contamination with pathogens, promoting the emergence of hyper-transmissible, and virulent pathogens. As a result, CAFOs have been associated with emergence of highly pathogenic avian influenza viruses, hepatitis E virus, *Escherichia coli* O157:H7, *Streptococcus suis*, livestock-associated methicillin-resistant *Staphylococcus aureus*, and *Cryptosporidium parvum* in farm animals. This has led to increased transmission of zoonotic pathogens in humans and changes in disease patterns in general communities. They are exemplified by the common occurrence of outbreaks of illnesses through direct and indirect contact with farm animals, and wide occurrence of similar serotypes or subtypes in both humans and farm animals in industrialized nations. Therefore, control measures should be developed to slow down the dispersal of zoonotic pathogens associated with CAFOs and prevent the emergence of new pathogens of epidemic and pandemic potential.

## Introduction of Concentrated Animal Feeding Operations

Humans have kept farm livestock and poultry ever since the domestication of various animals starting approximately 10,000 years ago (Diamond, 2002). For much of recorded history, humans have farmed these animals in traditional ways, keeping just enough animals for personal or family consumption (Salaheen et al., 2015). In these traditional systems, while humans are exposed to zoonotic pathogens because of possible introduction of pathogens from wildlife and intimate contact with animals (Pohjola et al., 2016; da Silva et al., 2018; Nicholson et al., 2020), the impact of such zoonotic transmission is limited by the small numbers of these animals (Espinosa et al., 2020).

To meet the growing demand for meat and other animal proteins after the end of the Second World War, animal farming in the Noth America and Europe transitioned from small-scale farming to concentrated animal feeding operations (CAFOs) (Graham et al., 2008). In general, they are animal facilities with over 700 mature dairy cattle, 1000 cow-calf pairs, 10000 sheep, 2500 adult swine, and 30000 laying hen or broilers^1^. This has led to not only changes in food animal production but also environmental and public health concerns about CAFOs (Kirkhorn, 2002). Initial public health concerns of CAFOs were on water quality issues, respiratory diseases among farmers and their neighbors, the spread of antimicrobial resistance, and global warming (Kirkhorn, 2002; Radon et al., 2007; Koneswaran and Nierenberg, 2008). As a result, various governmental regulations have been established in industrialized nations on CAFOs (Rosov et al., 2020).

Recent evidence suggests CAFOs may be contributing to significant changes in patterns of infectious diseases, with increased transmission of zoonotic pathogens (Moyer, 2016; Klumb et al., 2020). It has been suggested that the congregation of large numbers of susceptible animals in confined spaces and reduced genetic diversity of animals could promote the transmission of established pathogens in CAFOs (Jones et al., 2013). This can be mediated through the amplification and mutation of the pathogens, leading to their spread and the emergence of new variants with better adaptation to mammals and increased transmissibility and virulence (Espinosa et al., 2020). Data from one recent study suggest that since 1940, agricultural drivers were associated with >50% of zoonotic diseases in humans (Rohr et al., 2019).

**FIGURE 1:**
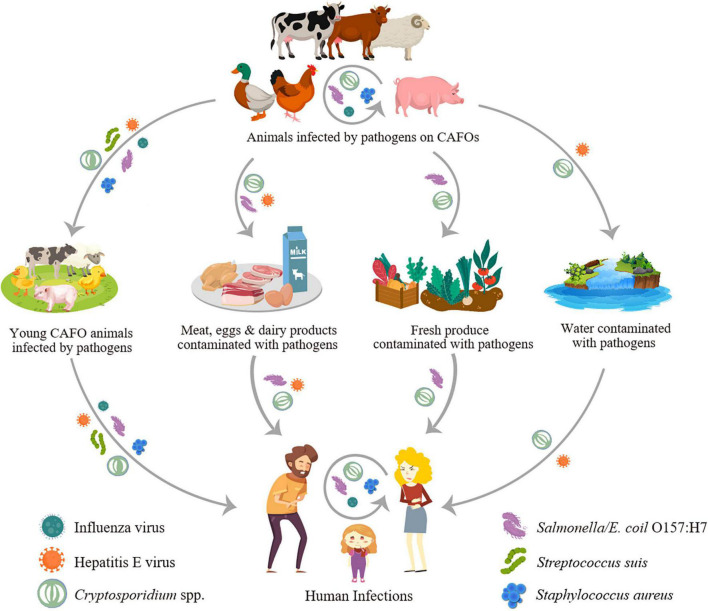
Transmission of major zoonotic pathogens through concentrated animal feeding operations (CAFOs).

Concentrated animal feeding operations have multiple ways to increase the transmission of zoonotic pathogens in humans (Figure 1). Farm animals may be infected with human pathogens such as *Salmonella*, *Campylobacter*, and *Cryptosporidium*, thus became reservoir or amplifier hosts and transmit them to humans through direct contact or contamination of food (meat, milk, eggs, and fresh produce, etc.) and drinking source water. They can also become the host allowing pathogens of wildlife origins such as avian influenza viruses to evolve with better adaptations to humans (Jones et al., 2013). The wide use of antibiotics in farm animals can also select for resistant pathogens such as livestock-associated methicillin-resistant *Staphylococcus aureus* (LA-MRSA) that have become a public health problem in European countries and some other areas (Sieber et al., 2018).

In this report, we have conducted a review of recent data on the association between the increased occurrence of representative zoonotic pathogens and CAFOs. These viral, bacterial, and parasitic pathogens are readily transmitted between animals and humans, although the present review focuses mostly on human infections acquired from animals. Several major zoonotic pathogens of public health importance in industrialized nations and with evidence on the effect of animal farming on increased pathogen transmission in humans are discussed (Jones et al., 2013). No efforts are made to summarize data extensively on environmental contamination of these pathogens by CAFOs due to the diverse transmission routes of these pathogens (Table 1).

**TABLE 1:** Major zoonotic pathogens associated with concentrated animal feeding operations (CAFOs) in industrialized nations.

Pathogen	Major CAFO type involved	Major risk factor for human infection	Epidemic potential	References
Influenza virus (H1N1, H5N1, and H7N9)	Swine, poultry	Animal contact	Outbreaks and pandemics	Nunez and Ross, 2019
Hepatitis E virus	Swine	Animal contact, foodborne, waterborne	Outbreaks	Geng and Wang, 2016
*Streptococcus suis*	Swine	Animal contact	Sporadic	Dutkiewicz et al., 2017
Livestock-associated methicillin-resistant *Staphylococcus aureus*	Swine	Animal contact	Sporadic	Sieber et al., 2018
*Salmonella* and *Campylobacter*	Cattle, poultry	Foodborne, animal contact	Outbreaks	Pogreba-Brown et al., 2018
*Escherichia coli* O157:H7	Cattle	Foodborne, animal contact	Outbreaks	Karmali, 2018
*Cryptosporidium parvum*	Dairy cattle	Animal contact, foodborne, waterborne	Outbreaks	Zahedi and Ryan, 2020

## Concentrated Animal Feeding Operations and Transmission of Major Viral Pathogens

Concentrated animal feeding operations have been associated with the transmission of several emerging zoonotic pathogens (Table 1). Such an association is probably best illustrated by the emergence of the pandemic influenza viruses. For example, duck farming provides the opportunity of spillover transmission of influenza A viruses from wild aquatic birds to chickens and pigs. This likely increases the occurrence of reassorted viruses with better infectivity to humans (Nunez and Ross, 2019). In addition, large pig and poultry farms have long been known as amplifiers of zoonotic influenza viruses (Henritzi et al., 2020). H1N1, H1N2, H3N2, and A(H1N1)pdm09 viruses are common in pigs in most countries around the world (Chauhan and Gordon, 2020), and several other avian influenza viruses such as H5N1 and H7N9 have emerged as important human pathogens in recent years (Philippon et al., 2020). It has been estimated that when CAFO workers comprise 15–45% of the community, the overall human influenza cases increase by 42–86% (Saenz et al., 2006).

Because of the prevalence of influenza A viruses in pigs and poultry, CAFO farmers are at significantly increased risk of influenza virus infection due to frequent contact with infected animals (Quan et al., 2019). Zoonotic transmission of influenza viruses has been commonly reported in swine farmers in Asian and European countries, mostly due to H1N1, especially A(H1N1)pdm09 (Chauhan and Gordon, 2020). In a recent longitudinal study conducted in China, swine workers at CAFOs had higher seroconversion to swine H1N1 and H3N2 than unexposed and non-CAFO swine workers (Borkenhagen et al., 2020). Another study showed increased seroprevalence of antibodies to H5N1, H7N9, and other subtypes in poultry workers, with some workers having seroconversion after employment on farms (Quan et al., 2019). The spillover of influenza viruses from CAFOs to the general community has been responsible for several recent influenza pandemics (Hollenbeck, 2016; Chauhan and Gordon, 2020), mostly caused by reassorted viruses (Dadonaite et al., 2019).

A particular concern is the reassortment of influenza A viruses in CAFOs. It is known that the colocation of swine and poultry farms, which is common in some areas and promotes the interspecies transmission of virus from birds to swine, can lead to the emergence of new influenza viruses (Nunez and Ross, 2019; Chauhan and Gordon, 2020). Pigs are known as a mixing vessel for influenza virus reassortment and evolution, as they can be infected by swine, avian and human influenza A viruses (Chauhan and Gordon, 2020). Avian-derived viruses may become adapted in pigs, facilitating the emergence of double- and triple-reassortant genotypes of pandemic potential (Chauhan and Gordon, 2020). For example, molecular surveillance of swine influenza viruses on pig farms has revealed intensive reassortment of viruses with A(H1N1)pdm09 virus, producing a repertoire of over 30 distinct genotypes of unknown virulence and tissue tropism (Henritzi et al., 2020). Poultry farms also play an important role in the evolution of highly pathogenic avian influenza viruses, leading to the introduction of influenza viruses of wild waterfowl into humans (Nunez and Ross, 2019; Liu et al., 2021).

Another viral pathogen on the rise in industrialized nations due to CAFOs is the hepatitis E virus (HEV). Although HEV infection used to be mainly endemic in low and middle-income countries, its prevalence has been steadily increasing in industrialized countries in recent years. This is largely due to zoonotic transmission of HEV from pigs in these countries (Christou and Kosmidou, 2013). Pigs are natural reservoirs of HEV and mostly show no clinical signs of infection (Sooryanarain and Meng, 2020). Seroprevalence of HEV is generally higher than 40% in pigs in most industrialized nations and China (Sooryanarain and Meng, 2020; Chen et al., 2021).

Human HEV infections in industrialized nations are exclusively caused by zoonotic genotypes 3 and 4 that circulate in pigs and wild mammals, while those in low and middle-income countries are caused by genotypes 1 and 2 through poor hygiene and contaminated water (Pallerla et al., 2020; Table 2). Therefore, HEV infections in industrialized nations are often attributed to occupational exposures to pigs and consumption of under-cooked pork and other animal products (De Schryver et al., 2015; Teixeira et al., 2017). Waterborne transmission of HEV is a potential concern, as contamination of raw source water from swine CAFOs occurs often (Gentry-Shields et al., 2015; La Rosa et al., 2017).

**TABLE 2:** Differences in the transmission of hepatitis E virus genotypes in humans around the world*.

Characteristics	Genotype of hepatitis E virus			
	1	2	3	4
Distribution	Africa and Asia	Mexico, West Africa	Industrialized nations	Asia
Major host	Human	Human	Pig, wild boar, deer, and rabbit	Pig, wild boar, and ruminants
Zoonotic transmission	No	No	Yes	Yes
Transmission route	Waterborne	Waterborne	Foodborne	Foodborne
Susceptible population	Young adults	Young adults	Older adults, immuno-compromised persons	Young adults
Chronic infection	No	No	Yes	No
Occurrence of outbreaks	Common	Localized	Occasional	Occasional

*Adapted from https://www.cdc.gov/hepatitis/hev/hevfaq.htm#section2.

In China, as pig farming intensifies, the molecular epidemiology of hepatitis E has evolved from high endemicity of genotype 1 associated with waterborne transmission toward low endemicity of genotype 4 in association with foodborne transmission due to undercooked pork or seafood products such as shellfish (Geng and Wang, 2016; Sridhar et al., 2017). A recent survey of pigs in seven provinces in China has shown a high seroprevalence of 67.1% with no apparent geographic and age differences (Zhou et al., 2019). In another meta-analysis of data from pigs, the seroprevalence was 48.0% and the prevalence of viral RNA was 14.4% (Chen et al., 2021). In humans, IgG against HEV was detected in 26.2% of general population and 48.4% of occupational workers with exposures to pigs (Shu et al., 2019). This is similar to results of a meta-analysis of HEV studies in China, which showed seroprevalence of 27.3 and 47.4% in the general population and occupational population, respectively (Yue et al., 2019). Unlike the detection of genotype 1 in historic isolates, most recent human and swine isolates from China belonged to genotype 4 (Li et al., 2019; Shu et al., 2019; Zhou et al., 2019). However, genotype 3, which is common in industrialized nations, has been identified recently in several species of animals and raw pork livers in China, suggesting its likely emergence in humans in the near future (Go et al., 2019; Rui et al., 2020).

## Concentrated Animal Feeding Operations and Transmission of Major Bacterial Pathogens

Concentrated animal feeding operations have possibly played an important role in the emergence of foodborne and animal-contact associated bacterial pathogens in industrialized nations (Table 1). Dairy cattle are well-known reservoirs of *Escherichia coli* O157:H7, non-typhoidal *Salmonella*, and *Campylobacter* (NASPHV, 2011; Levallois et al., 2014; Palomares Velosa et al., 2020). Large poultry farms with poor rearing hygiene are further known to have high prevalence of *Salmonella* and *Campylobacter* (Koutsoumanis et al., 2019; Seman et al., 2020). As a result, occupational and agricultural exposures have been identified as key risk factors for human infections with these pathogens in industrialized nations (Levallois et al., 2014; Conrad et al., 2017; Su et al., 2017; Pogreba-Brown et al., 2018; Klumb et al., 2020). Outbreaks of salmonellosis, campylobacteriosis, and *E. coli* O157:H7 infections have been reported in association with direct contact of animals (calves, lambs, goat kids, and live poultry, etc.) on or from CAFOs in industrialized nations (Conrad et al., 2017; Marus et al., 2019; Table 1). These pathogens are the most common causes of foodborne outbreaks of illnesses in the United States (Tack et al., 2020).

It has been suggested that CAFOs have played a crucial role in the emergence of the highly pathogenic *E. coli* O157:H7 in the 1980s in industrialized nations (Karmali, 2018). The hemolytic uremic syndrome (HUS) induced by O157:H7 has the highest incidence in Europe, North America, Argentina, Australia, New Zealand, and Japan, where cattle farming is most intensive (Franz et al., 2019). Phylogenetic timing of whole genome sequence data indicates that the initial emergence of the highly pathogenic O157:H7 from non-pathogenic *E. coli* serotype O55:H7 might have occurred in Netherlands over 130 years ago (Dallman et al., 2015). The diversification of O157:H7, however, occurred much later, with the dissemination of various clades around the world mostly during the last 30–50 years, due to the movement of Holstein-Friesian dairy cattle in association with the emergence of CAFOs (Dallman et al., 2015; Franz et al., 2019). The incidence of human cases of *E. coli* O157:H7 correlates with cattle density and cattle to human ratios (Saeedi et al., 2017). As a result, farm visiting is a key risk factor for human infection with O157:H7 in some areas (Money et al., 2010), and the highest incidence of HUS, the most severe clinical manifestation caused by O157:H7, has been reported in North America, Europe, and Japan (Saeedi et al., 2017; Franz et al., 2019). It could be argued that this could be due to the better surveillance of the disease in industrialized nation. Nevertheless, the incidence of HUS is very low in China, where surveillance program for infectious diseases is vagarous since the SARS outbreak in 2003 (Chen et al., 2014).

Other emerging bacterial pathogens in humans in association with CAFOs include *Streptococcus suis* and *Staphylococcus aureus* (Filippitzi et al., 2017). *S. suis* is a commensal bacterium of tonsils and nasal cavities in young pigs, but can cause meningitis and sepsis in humans. Human cases of *S. suis* infection have been on the rise in several countries, especially those in Asia (Huong et al., 2019; Susilawathi et al., 2019; Gajdacs et al., 2020; Jiang et al., 2020; Kerdsin et al., 2020). Most of the cases have had occupational exposures to pigs, usually through contamination of minor cuts or abrasions on skin or by pig bite (Dutkiewicz et al., 2017). Among the numerous serotypes in pigs, serotype 2, especially its Sequence Type 1 (ST1), is the most common serotype for human infections (Dutkiewicz et al., 2017; Susilawathi et al., 2019; Agoston et al., 2020). It was responsible for two outbreaks in China (Dong et al., 2021). Humans in China are also infected with serotype 2, ST7 (Wang et al., 2019; Jiang et al., 2020).

A recent phylogenomic analysis on 1,634 *S. suis* isolates from 14 countries over 36 years has identified a novel human-associated clade (HAC) divergent from the diseased-pig clade (DPC) and healthy-pig clade (HPC). HAC appeared to have originated from Europe through the export of European swine breeds between 1960s and 1970s (Dong et al., 2021). Within HAC, ST7 is the China-specific virulent variant in lineages I and III while ST1 belongs to lineage II and is the most common sequence type in HAC. ST1 isolates from humans in China are closely related to those from Vietnam (Dong et al., 2021).

Livestock-associated methicillin-resistant *S. aureus* (LA-MRSA) clonal complex 398 (CC398 or ST398) is another emerging zoonotic pathogen in industrialized nations, especially those in Europe (Larsen et al., 2017). A high colonization rate (21.6%) of MRSA was seen in swine farm workers from a survey conducted in Italy. Almost all human MRSA isolates had the same sequence type obtained from pigs on the farm (Pirolo et al., 2019). Import of pathogens from other European countries had a major impact on the emergence of LA-MRSA CC398 in Italy, but local trading of pigs among farms also played an important role in the dissemination of the pathogen (Pirolo et al., 2020). There has been a recent increase in the transmission of the pathogen in farm animals; a study in Switzerland had shown a dramatic increase in LA-MRSA prevalence in pigs, from 2% in 2009 to 44% in 2017. In Germany, the prevalence of LA-MRSA CC398 in humans is much higher in regions with intensive animal farming (Kock et al., 2014). Whole-genome analysis showed sequence similarity between isolates from farmers and their pigs (Kittl et al., 2020).

Denmark, Netherlands, and Slovenia have experienced a significant increase in human infections with LA-MRSA CC398 in recent years (Kinross et al., 2017; Sieber et al., 2018; Dermota et al., 2020). Results of meta-analysis indicate that livestock workers, particularly swine farmers, are at significantly higher risk for LA-MRSA infection (Chen and Wu, 2020). Many of the cases, however, had no direct contact with livestock but tended to live in rural areas, suggesting the likely occurrence of secondary transmission of the pathogen in the community (Larsen et al., 2017; Sieber et al., 2019). In agreement with this, LA-MRSA CC398 appears to be spreading to other domesticated animals such as veal calves, horses and farmed minks in Europe (Aires-de-Sousa, 2017; Albert et al., 2019; Hansen et al., 2020; Lienen et al., 2021). It has been demonstrated that LA-MRSA CC398 may spread among animals, humans, and the environment on dairy farms (Lienen et al., 2021).

The geographic range of LA-MRSA ST398 is expanding. It has been reported in pigs and humans in recent years in Korean and Japan (Lee et al., 2021; Nakaminami et al., 2021; Sasaki et al., 2021). LA-MRSA ST398 isolates were recovered from milk samples from two farms in China in one study. They had genomes closely related to the human isolates in the country (Cui et al., 2020). However, sequence type 9 is the most common LA-MRSA in humans and pigs in China and other Asian countries, probably because ST398 has not been dispersed widely yet (Yu et al., 2021).

## Concentrated Animal Feeding Operations and Transmission of Zoonotic Parasite *Cryptosporidium Parvum*

Like in viral and bacterial pathogens, farming activities also have extensive impact on the transmission of parasites. A recent study has suggested that animal farming and trading have influenced the evolution and transmission of *Toxoplasma gondii* significantly (Shwab et al., 2018). Similarly, the housing of large numbers of chickens on modern poultry farms facilitates the transmission of *Eimeria* spp., making the prevention and control of coccidiosis a heavy burden to farmers (Fatoba and Adeleke, 2018). The improved housing and waste disposal in CAFOs have in general reduced the transmission of soil transmitted helminths such as *Ascaris suum*, *Strongyloides ransomi*, and *Trichuris suis* in pigs (Symeonidou et al., 2020). The best example for the impact of CAFOs on the transmission of zoonotic parasites and their public health importance, however, is *Cryptosporidium parvum*.

*Cryptosporidium parvum* is particularly common on dairy farms, with virtually all farms examined in industrialized nations being positive for *C. parvum* (Diaz et al., 2018; Razakandrainibe et al., 2018; Lombardelli et al., 2019; Santoro et al., 2019). On these farms, *C. parvum* infection in neonatal calves starts soon after birth, peaks in calves of 1–2 weeks of age, with almost all calves acquiring infection before weaning around 8 weeks (Thomson et al., 2019). *C. parvum*, however, is rarely seen in weaned calves and older animals, which are mostly infected with *C. bovis*, *C. ryanae*, and *C. andersoni* (Santin, 2020). The latter species are less pathogenic and largely non-infective to humans. With a few exceptions, *C. parvum* infections in dairy calves in industrialized nations are mostly caused by the IIa subtypes (Feng et al., 2018).

*Cryptosporidium parvum* is less frequently seen in calves raised in less intensive production systems. In Sweden, where dairy farming is much less intensive than other European countries, *C. parvum* is rarely seen in healthy pre-weaned calves, although it is the dominant species in calves that died of diarrhea (Silverlas and Blanco-Penedo, 2013; Silverlas et al., 2013). In beef cattle, which are usually managed using the traditional cow-calf grazing system, several molecular epidemiological studies have identified *C. bovis* and *C. ryanae* as the dominant species in industrialized nations (Rieux et al., 2013; Bjorkman et al., 2015). Thus, in less intensively managed production systems, *C. parvum* is either absent or only seen in calves with diarrhea (Kabir et al., 2020).

Because of the low intensive nature of animal farming, native calves in low- and middle-income countries are rarely infected with *C. parvum*. Results of studies in African, Asian, and South American countries have shown a dominance of *C. bovis* and *C. ryanae* in native calves with *C. parvum* largely absent (Ayinmode et al., 2010; Maikai et al., 2011; Nguyen et al., 2012; Abu Samra et al., 2013). This is also mostly the case with other bovine animals such as yaks and water buffaloes (Martins et al., 2018; Ren et al., 2019; Wu et al., 2020). *C. ryanae* is especially common in water buffaloes (de Aquino et al., 2020; Russell et al., 2020).

The high prevalence of *C. parvum* in dairy cattle in industrialized nations has led to spillover infections in other farm animals, probably through sharing pastures and drinking contaminated water. This is supported by the occurrence of *C. parvum* in lambs and goat kids in European countries. While *C. xiaoi* and *C. ubiquitum* are the dominant species in lambs in Asian and African countries, *C. parvum* is the dominant species in lambs in many European countries (Guo et al., 2021). The same IIa subtypes circulating in calves there also circulate in lambs (Guo et al., 2021). In northeastern Spain, however, lambs are mostly infected with IId subtypes (Quilez et al., 2008). A similar distribution in *Cryptosporidium* species is also seen in goats; *C. parvum* IIa subtypes are common in goat kids in Europe (Guo et al., 2021). Outside Europe, only some Middle East and Northern African countries, Australia and possibly New Zealand are known to have moderate occurrence of *C. parvum* in lambs and goat kids (Hijjawi et al., 2016; Al-Habsi et al., 2017; Baroudi et al., 2018; Majeed et al., 2018; Sahraoui et al., 2019).

The high prevalence of *C. parvum* in ruminants appear to have significant impacts on cryptosporidiosis epidemiology in some industrialized nations where CAFOs are a common presence. For example, contact with farm animals, especially calves and lambs, is a major risk factor for cryptosporidiosis occurrence in humans in the United States, Europe, and New Zealand (Nic Lochlainn et al., 2019; Costa et al., 2020; Garcia et al., 2020; Loeck et al., 2020; Table 1). In a recent analysis of surveillance data collected during 2012–2016 in Minnesota, United States, 60% of *C. parvum* cases had reported animal exposures in their incubation period (Klumb et al., 2020). Outbreaks of cryptosporidiosis due to contact with infected animals frequently occur in veterinary students in some industrialized nations (Kinross et al., 2015; Benschop et al., 2017; Thomas-Lopez et al., 2020). Other outbreaks of zoonotic *C. parvum* infections have occurred in caretakers of sick calves, farm visitors, children attending agricultural camps, and emergency responders rescuing calves in traffic accidents and burning barns (Conrad et al., 2017).

**FIGURE 2:**
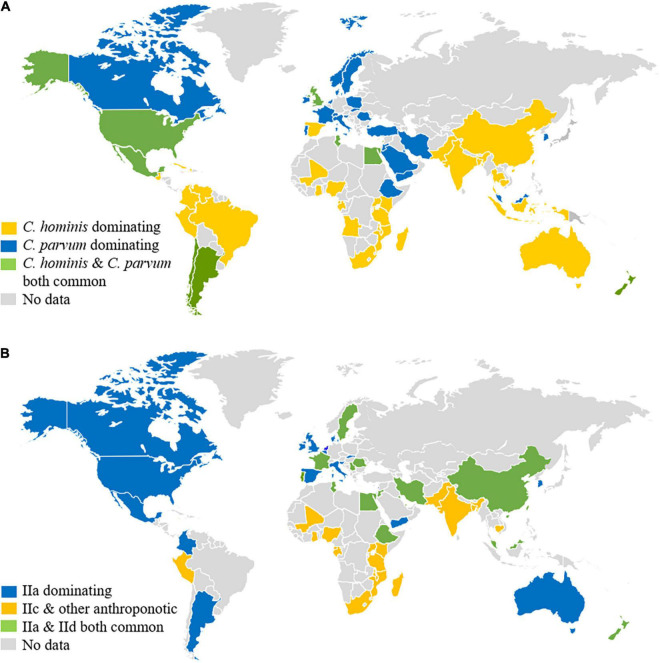
Distribution of major *Cryptosporidium* species **(A)** and *Cryptosporidium parvum* subtypes **(B)** in humans around the world. Original data are presented in Supplementary Tables 1, 2 and a recent review (Yang et al., 2021).

The high prevalence of *C. parvum* in CAFOs could have affected the infection patterns of *Cryptosporidium* spp. in humans. In low- and middle-income countries, *C. hominis*, which is an anthroponotic species, is the dominant *Cryptosporidium* species in humans (Yang et al., 2021; Figure 2A). This is also the case in some industrialized countries such as the United States, Japan, and Australia (Xiao, 2010). In European countries and New Zealand, however, *C. parvum* is responsible for over 50% of human *Cryptosporidium* infections (Costa et al., 2020; Garcia et al., 2020; Lebbad et al., 2021). Even in the United States and Australia where *C. hominis* dominates over *C. parvum* country-wide, *C. parvum* is the dominant species in humans in rural areas (Braima et al., 2019; Loeck et al., 2020). In these countries, *C. parvum* is responsible for numerous waterborne, foodborne, and animal contact-associated outbreaks of cryptosporidiosis in the general community (Insulander et al., 2013; Chalmers et al., 2019). Of particular concern is the common occurrence of waterborne outbreaks of *C. parvum*-associated cryptosporidiosis (Zahedi and Ryan, 2020), as this species is the dominant *Cryptosporidium* species in drinking source water in most industrialized nations (Swaffer et al., 2018; Ligda et al., 2020; Mphephu et al., 2021). Identical distribution of *Cryptosporidium* species has been found between farms animals and downstream surface water, supporting the contribution of CAFOs to environmental contamination of oocysts (Zahedi et al., 2020).

Genetic characterization of *C. parvum* in humans supports the role of CAFOs in the epidemiology of cryptosporidiosis. While *C. parvum* infections in low- and middle-income countries are mostly caused by the anthroponotic IIc subtype family (Yang et al., 2021), *C. parvum* infections in humans in industrialized nations are largely caused by the IIa subtype family, the zoonotic *C. parvum* commonly found in calves and lambs in areas practicing CAFOs (Xiao, 2010; Figure 2B). In Europe and New Zealand, the distribution of *C. parvum* IIa subtypes has been virtually identical between humans and calves. The peak occurrence of human *C. parvum* infections by these subtypes coincides with the spring calving and lambing (Garcia et al., 2020; O’Leary et al., 2020). Similarly, human infections with IId subtypes are only found in countries where these subtypes occur in calves, lambs, and goat kids, such as Europe, the Middle East, China, and New Zealand (Figure 2B). The genetic similarities observed between the human and bovine isolates suggest that dairy calves are significant amplifiers of zoonotic *C. parvum* subtypes (Al Mawly et al., 2015).

A major public health consequence of the common occurrence of *C. parvum* in farm animals is the frequent association of this species with outbreaks of human cryptosporidiosis in industrialized nations. In the United Kingdom, over half of the cryptosporidiosis outbreaks are caused by *C. parvum* (Chalmers et al., 2019). Outbreaks by *C. parvum* are also common in other industrialized nations such as other European countries, the United States, and Australia (Caccio and Chalmers, 2016; Xiao and Feng, 2017; Zahedi and Ryan, 2020). Most of the outbreaks were animal contact-associated or foodborne, while those caused by *C. hominis* were associated with recreational water (Chalmers et al., 2019). One *C. parvum* subtype, IIaA15G2R1, is particularly virulent, having been the dominant *C. parvum* subtypes for outbreaks in Europe and United States (Xiao and Feng, 2017; Chalmers et al., 2019). It is also the dominant *C. parvum* subtype in dairy calves in most industrialized nations (Xiao, 2010). As a result, outbreaks of cryptosporidiosis by this subtype and other subtypes are common in veterinary students in these countries (Thomas-Lopez et al., 2020).

## Public Health Perspectives and Conclusion

Data accumulated thus far suggest that CAFOs have significant impacts on the transmission of some viral, bacterial, and parasitic pathogens in some areas, especially *C. parvum* and HEV. They provide an ecological niche that promotes the emergence of virulent and hyper-transmissible pathogens via the congregation of susceptible animals in confined spaces, reduced genetic diversity of the host, and increased animal movement across farms and other commercial operations (such as the import of high-performance breeds and constant transfer of male dairy calves to veal and beef farms) (Saenz et al., 2006; Jones et al., 2013; Hollenbeck, 2016). Some modern living practices select zoonotic pathogens with better adaptation to the human-animal interface and specific transmission routes, such as highly pathogenic influenza virus, HEV, *E. coli* O157:H7 serotype, *S. suis*, LA-MRSA, and *C. parvum* (Engering et al., 2013; Espinosa et al., 2020). They include wide use of antibiotics, treating drinking source water with chlorine, increased consumption of unpasteurized milk and juices, undercooked meat and raw fresh produce, and occupational and educational exposures to farm animals. These environmental and culture factors promote spillover infections in the general communities and epidemic disease outbreaks (Figure 1). While these impacts are mostly seen in industrialized nations where animal production is most intensive, they start to influence pathogen transmission in countries where CAFOs are in early implementation. This is exemplified by the increased transmission of zoonotic genotypes 3 and 4 of HEV and the emergence of *S. suis* infections in humans in China.

As the practice of CAFOs has only a short history, we are probably yet to see its full public health impacts. Studies conducted thus far indicate that countries having the highest O157:H7 incidence in humans also have high prevalence of *C. parvum* IIa subtypes in dairy calves, such as North America, Europe, Japan, Australia, and New Zealand (Xiao, 2010; Franz et al., 2019). Both pathogens are responsible for significant numbers of foodborne and animal contact-associated outbreaks in these areas (Conrad et al., 2017; Karmali, 2018; Zahedi and Ryan, 2020). While the public health impacts of CAFOs on the transmission of *C. parvum* is more obvious in European countries and New Zealand, such effects appear to be focal in other industrialized nations, with *C. parvum* IIa subtypes inducing significant disease burdens in humans mostly in rural areas (Xiao, 2010; Loeck et al., 2020). Similarly, *S. suis* and LA-MRSA CC398 are increasing in distribution and public health significance. Therefore, control measures should be implemented to prevent the emergence of zoonotic pathogens as major public health problems in these areas.

Public health measures should also be implemented to prevent the emergence of new zoonotic pathogens in CAFOs. In addition to the known pathogens discussed above, surveillance should be installed to monitor the transmission of other potential zoonotic pathogens that have broad host range, undergo through frequent recombination for better adaptation to humans, or can easily acquire drug resistance genes through mobile genetic elements. The association between the prevalence of some antibiotic resistance genes in foodborne bacteria and drug usage in farm animals is well established (Luiken et al., 2019). Therefore, other LA-MRSA clonal complexes and resistant bacteria derived from farm animals could become future public health problems. Of more immediate concern is the possible spillover of SARS-CoV-2 from humans to farm animals, as the virus has been shown to be infective to several species of farm and companion animals (Shi et al., 2020), and has known to cause epidemics in farmed minks and wild white-tailed deer (Chandler et al., 2021; Lu et al., 2021). If it manages to infect farm animals, the large number of animals in CAFOs will likely complicate current control efforts against COVID-19. Therefore, molecular surveillance of SARS-CoV-2 and other zoonotic pathogens in CAFOs, such as pathogen discovery using metagenomic data from animal samples (Kawasaki et al., 2021), should be implemented for the forecast and prevention of new zoonotic pathogens of public health significance.

## Author Contributions

YF and LX secured funds for the work. YG, UR, YF, and LX conducted data collection and analysis. YG and LX prepared the draft manuscript. UR and LX edited the final manuscript. All authors contributed to the article and approved the submitted version.

## Conflict of Interest

The authors declare that the research was conducted in the absence of any commercial or financial relationships that could be construed as a potential conflict of interest.

## Publisher’s Note

All claims expressed in this article are solely those of the authors and do not necessarily represent those of their affiliated organizations, or those of the publisher, the editors and the reviewers. Any product that may be evaluated in this article, or claim that may be made by its manufacturer, is not guaranteed or endorsed by the publisher.

## Footnotes

1
https://www.epa.gov/sites/default/files/2015-08/documents/sector_table.pdf

## References

[B1] Abu Samra N. Jori F. Xiao L. H. Rikhotso O. Thompson P. N. (2013). Molecular characterization of *Cryptosporidium* species at the wildlife/livestock interface of the Kruger National Park. South Africa. *Comp. Immunol. Microbiol. Infect. Dis.* 36 295–302. 10.1016/j.cimid.2012.07.004 22975725

[B2] Agoston Z. Terhes G. Hannauer P. Gajdacs M. Urban E. (2020). Fatal case of bacteremia caused by *Streptococcus suis* in a splenectomized man and a review of the European literature. *Acta Microbiol. Imunol. Hungarica* 67 148–155. 10.1556/030.2020.01123 32223305

[B3] Aires-de-Sousa M. (2017). Methicillin-resistant *Staphylococcus aureus* among animals: current overview. *Clin. Microbiol. Infect.* 23 373–380. 10.1016/j.cmi.2016.11.002 27851997

[B4] Al-Habsi K. Yang R. Williams A. Miller D. Ryan U. Jacobson C. (2017). Zoonotic *Cryptosporidium* and *Giardia* shedding by captured rangeland goats. *Vet. Parasitol. Reg. Stud. Rep.* 7 32–35. 10.1016/j.vprsr.2016.11.006 31014653

[B5] Al Mawly J. Grinberg A. Velathanthiri N. French N. (2015). Cross sectional study of prevalence, genetic diversity and zoonotic potential of *Cryptosporidium parvum* cycling in New Zealand dairy farms. *Parasit. Vectors* 8:240. 10.1186/s13071-015-0855-9 25896433 PMC4423479

[B6] Albert E. Biksi I. Nemet Z. Csuka E. Kelemen B. Morvay F. (2019). Outbreaks of a methicillin-resistant *Staphylococcus aureus* clone ST398-t011 in a Hungarian equine clinic: Emergence of rifampicin and chloramphenicol resistance after treatment with these antibiotics. *Microb. Drug Resist.* 25 1219–1226. 10.1089/mdr.2018.0384 31066624

[B7] Ayinmode A. B. Olakunle F. B. Xiao L. (2010). Molecular characterization of *Cryptosporidium* spp. in native calves in Nigeria. *Parasitol. Res.* 107 1019–1021. 10.1007/s00436-010-1972-1 20644959

[B8] Baroudi D. Hakem A. Adamu H. Amer S. Khelef D. Adjou K. (2018). Zoonotic *Cryptosporidium* species and subtypes in lambs and goat kids in Algeria. *Parasit. Vectors* 11:582. 10.1186/s13071-018-3172-2 30400983 PMC6219180

[B9] Benschop J. Booker C. M. Shadbolt T. Weston J. F. (2017). A retrospective cohort study of an outbreak of cryptosporidiosis among veterinary students. *Vet. Sci.* 4:29. 10.3390/vetsci4020029 29056688 PMC5606607

[B10] Bjorkman C. Lindstrom L. Oweson C. Ahola H. Troell K. Axen C. (2015). *Cryptosporidium* infections in suckler herd beef calves. *Parasitology* 142 1108–1114. 10.1017/S0031182015000426 25899555 PMC4453919

[B11] Borkenhagen L. K. Wang G. L. Simmons R. A. Bi Z. Q. Lu B. Wang X. J. (2020). High risk of influenza virus infection among swine workers: examining a dynamic cohort in China. *Clin. Infect. Dis.* 71 622–629. 10.1093/cid/ciz865 31504322 PMC7108185

[B12] Braima K. Zahedi A. Oskam C. Reid S. Pingault N. Xiao L. (2019). Retrospective analysis of *Cryptosporidium* species in Western Australian human populations (2015-2018), and emergence of the *C. hominis* IfA12G1R5 subtype. *Infect. Genet. Evol.* 73 306–313. 10.1016/j.meegid.2019.05.018 31146044

[B13] Caccio S. M. Chalmers R. M. (2016). Human cryptosporidiosis in Europe. *Clin. Microbiol. Infect.* 22 471–480. 10.1016/j.cmi.2016.04.021 27172805

[B14] Chalmers R. M. Robinson G. Elwin K. Elson R. (2019). Analysis of the *Cryptosporidium* spp. and gp60 subtypes linked to human outbreaks of cryptosporidiosis in England and Wales, 2009 to 2017. *Parasit. Vectors* 12:95. 10.1186/s13071-019-3354-6 30867023 PMC6417012

[B15] Chandler J. C. Bevins S. N. Ellis J. W. Linder T. J. Tell R. M. Jenkins-Moore M. (2021). SARS-CoV-2 exposure in wild white-tailed deer (Odocoileus virginianus). *Proc. Natl. Acad. Sci. USA* 118:e2114828118. 10.1073/pnas.2114828118 34732584 PMC8617405

[B16] Chauhan R. P. Gordon M. L. (2020). A systematic review analyzing the prevalence and circulation of influenza viruses in swine population worldwide. *Pathogens* 9:355. 10.3390/pathogens9050355 32397138 PMC7281378

[B17] Chen C. Wu F. (2020). Livestock-associated methicillin-resistant *Staphylococcus aureus* (LA-MRSA) colonisation and infection among livestock workers and veterinarians: a systematic review and meta-analysis. *Occup. Environ. Med.* 2020:106418. 10.1136/oemed-2020-106418 33097674

[B18] Chen Y. Chen X. Zheng S. Yu F. Kong H. Yang Q. (2014). Serotypes, genotypes and antimicrobial resistance patterns of human diarrhoeagenic *Escherichia coli* isolates circulating in southeastern China. *Clin. Microbiol. Infect.* 20 52–58. 10.1111/1469-0691.12188 23521436

[B19] Chen Y. Gong Q. L. Wang Q. Wang W. Wei X. Y. Jiang J. (2021). Prevalence of hepatitis E virus among swine in China from 2010 to 2019: a systematic review and meta-analysis. *Microb. Pathog.* 150:104687. 10.1016/j.micpath.2020.104687 33301857

[B20] Christou L. Kosmidou M. (2013). Hepatitis E virus in the Western world–a pork-related zoonosis. *Clin. Microbiol. Infect.* 19 600–604. 10.1111/1469-0691.12214 23594199

[B21] Conrad C. C. Stanford K. Narvaez-Bravo C. Callaway T. McAllister T. (2017). Farm fairs and petting zoos: a review of animal contact as a source of zoonotic enteric disease. *Foodborne Pathog. Dis.* 14 59–73. 10.1089/fpd.2016.2185 27992253

[B22] Costa D. Razakandrainibe R. Valot S. Vannier M. Sautour M. Basmaciyan L. (2020). Epidemiology of cryptosporidiosis in France from 2017 to 2019. *Microorganisms* 8:1358. 10.3390/microorganisms8091358 32899825 PMC7563450

[B23] Cui M. Li J. Ali T. Kalim K. Wang H. Song L. (2020). Emergence of livestock-associated MRSA ST398 from bulk tank milk, China. *J. Antimicrob. Chemother.* 75 3471–3474. 10.1093/jac/dkaa367 32797238

[B24] da Silva M. S. Silveira S. Caron V. S. Mosena A. C. S. Weber M. N. Cibulski S. P. (2018). Backyard pigs are a reservoir of zoonotic hepatitis E virus in southern Brazil. *Trans. R Soc. Trop. Med. Hyg.* 112 14–21. 10.1093/trstmh/try017 29554365

[B25] Dadonaite B. Gilbertson B. Knight M. L. Trifkovic S. Rockman S. Laederach A. (2019). The structure of the influenza A virus genome. *Nat. Microbiol.* 4 1781–1789. 10.1038/s41564-019-0513-7 31332385 PMC7191640

[B26] Dallman T. J. Ashton P. M. Byrne L. Perry N. T. Petrovska L. Ellis R. (2015). Applying phylogenomics to understand the emergence of Shiga-toxin-producing *Escherichia coli* O157:H7 strains causing severe human disease in the UK. *Microb. Genom.* 1:e000029. 10.1099/mgen.0.000029 28348814 PMC5320567

[B27] de Aquino M. C. C. Inacio S. V. Rodrigues F. S. de Barros L. D. Garcia J. L. Headley S. A. (2020). Cryptosporidiosis and *Giardia*sis in buffaloes (*Bubalus bubalis*). *Front. Vet. Sci.* 7:557967. 10.3389/fvets.2020.557967 33330686 PMC7673452

[B28] De Schryver A. De Schrijver K. Francois G. Hambach R. van Sprundel M. Tabibi R. (2015). Hepatitis E virus infection: an emerging occupational risk? *Occup. Med.* 65 667–672. 10.1093/occmed/kqv154 26452392

[B29] Dermota U. Kosnik I. G. Janezic S. Rupnik M. (2020). Changing epidemiology of presumptive community-associated-methicillin-resistant *Staphylococcus aureus* in Slovenia in 2014-2015 compared to 2010. *Zdr. Varst.* 59 236–244. 10.2478/sjph-2020-0030 33133280 PMC7583430

[B30] Diamond J. (2002). Evolution, consequences and future of plant and animal domestication. *Nature* 418 700–707. 10.1038/nature01019 12167878

[B31] Diaz P. Varcasia A. Pipia A. P. Tamponi C. Sanna G. Prieto A. (2018). Molecular characterisation and risk factor analysis of *Cryptosporidium* spp. in calves from Italy. *Parasitol. Res.* 117 3081–3090. 10.1007/s00436-018-6000-x 30008134 PMC7088234

[B32] Dong X. Chao Y. Zhou Y. Zhou R. Zhang W. Fischetti V. A. (2021). The global emergence of a novel *Streptococcus suis* clade associated with human infections. *EMBO Mol. Med.* 13:e13810. 10.15252/emmm.202013810 34137500 PMC8261479

[B33] Dutkiewicz J. Sroka J. Zajac V. Wasinski B. Cisak E. Sawczyn A. (2017). *Streptococcus suis*: a re-emerging pathogen associated with occupational exposure to pigs or pork products. Part I - Epidemiology. *Ann. Agric. Environ. Med.* 24 683–695. 10.26444/aaem/79813 29284248

[B34] Engering A. Hogerwerf L. Slingenbergh J. (2013). Pathogen-host-environment interplay and disease emergence. *Emerg. Microbes Infect.* 2:e5. 10.1038/emi.2013.5 26038452 PMC3630490

[B35] Espinosa R. Tago D. Treich N. (2020). Infectious diseases and meat production. *Environ. Resour. Econ.* 2020 1–26. 10.1007/s10640-020-00484-3 32836843 PMC7399585

[B36] Fatoba A. J. Adeleke M. A. (2018). Diagnosis and control of chicken coccidiosis: a recent update. *J. Parasit. Dis.* 42 483–493. 10.1007/s12639-018-1048-1 30538344 PMC6261147

[B37] Feng Y. Ryan U. M. Xiao L. (2018). Genetic diversity and population structure of *Cryptosporidium*. *Trends Parasitol.* 34 997–1011. 10.1016/j.pt.2018.07.009 30108020

[B38] Filippitzi M. E. Goumperis T. Robinson T. Saegerman C. (2017). Microbiological zoonotic emerging risks, transmitted between livestock animals and humans (2007-2015). *Transbound. Emerg. Dis.* 64 1059–1070. 10.1111/tbed.12484 28670863

[B39] Franz E. Rotariu O. Lopes B. S. MacRae M. Bono J. L. Laing C. (2019). Phylogeographic analysis reveals multiple international transmission events have driven the global emergence of *Escherichia coli* O157:H7. *Clin. Infect. Dis.* 69 428–437. 10.1093/cid/ciy919 30371758

[B40] Gajdacs M. Nemeth A. Knausz M. Barrak I. Stajer A. Mestyan G. (2020). *Streptococcus suis*: an underestimated emerging pathogen in Hungary? *Microorganisms* 8:1292. 10.3390/microorganisms8091292 32847011 PMC7570012

[B41] Garcia R. J. Pita A. B. Velathanthiri N. French N. P. Hayman D. T. S. (2020). Species and genotypes causing human cryptosporidiosis in New Zealand. *Parasitol. Res.* 119 2317–2326. 10.1007/s00436-020-06729-w 32494897

[B42] Geng Y. Wang Y. (2016). Epidemiology of Hepatitis E. *Adv. Exp. Med. Biol.* 948 39–59. 10.1007/978-94-024-0942-0_3 27738978

[B43] Gentry-Shields J. Myers K. Pisanic N. Heaney C. Stewart J. (2015). Hepatitis E virus and coliphages in waters proximal to swine concentrated animal feeding operations. *Sci. Total Environ.* 505 487–493. 10.1016/j.scitotenv.2014.10.004 25461050 PMC4514618

[B44] Go H. J. Park B. J. Ahn H. S. Lyoo E. L. Kim D. H. Lee J. B. (2019). Identification of hepatitis E virus in bovine and porcine raw livers. *J. Microbiol. Biotechnol.* 29 2022–2025. 10.4014/jmb.1910.10059 31752068

[B45] Graham J. P. Leibler J. H. Price L. B. Otte J. M. Pfeiffer D. U. Tiensin T. (2008). The animal-human interface and infectious disease in industrial food animal production: rethinking biosecurity and biocontainment. *Public Health Rep.* 123 282–299. 10.1177/003335490812300309 19006971 PMC2289982

[B46] Guo Y. Li N. Ryan U. Feng Y. Xiao L. (2021). Small ruminants and zoonotic cryptosporidiosis. *Parasitol. Res.* 120 4189–4198. 10.1007/s00436-021-07116-9 33712929

[B47] Hansen J. E. Stegger M. Pedersen K. Sieber R. N. Larsen J. Larsen G. (2020). Spread of LA-MRSA CC398 in Danish mink (*Neovison vison*) and mink farm workers. *Vet. Microbiol.* 245:108705. 10.1016/j.vetmic.2020.108705 32456821

[B48] Henritzi D. Petric P. P. Lewis N. S. Graaf A. Pessia A. Starick E. (2020). Surveillance of European domestic pig populations identifies an emerging reservoir of potentially zoonotic swine influenza a Viruses. *Cell Host Mcrobe.* 28:e616. 10.1016/j.chom.2020.07.006 32721380

[B49] Hijjawi N. Mukbel R. Yang R. Ryan U. (2016). Genetic characterization of *Cryptosporidium* in animal and human isolates from Jordan. *Vet. Parasitol.* 228 116–120. 10.1016/j.vetpar.2016.08.015 27692311

[B50] Hollenbeck J. E. (2016). Interaction of the role of concentrated animal feeding operations (CAFOs) in emerging infectious diseases (EIDS). *Infect. Genet. Evol.* 38 44–46. 10.1016/j.meegid.2015.12.002 26656834 PMC7106093

[B51] Huong V. T. L. Turner H. C. Kinh N. V. Thai P. Q. Hoa N. T. Horby P. (2019). Burden of disease and economic impact of human *Streptococcus suis* infection in Viet Nam. *Trans. R Soc. Trop. Med. Hyg.* 113 341–350. 10.1093/trstmh/trz004 30809669 PMC6580695

[B52] Insulander M. Silverlas C. Lebbad M. Karlsson L. Mattsson J. G. Svenungsson B. (2013). Molecular epidemiology and clinical manifestations of human cryptosporidiosis in Sweden. *Epidemiol. Infect.* 141 1009–1020. 10.1017/S0950268812001665 22877562 PMC9151846

[B53] Jiang F. Guo J. Cheng C. Gu B. (2020). Human infection caused by *Streptococcus suis* serotype 2 in China: report of two cases and epidemic distribution based on sequence type. *BMC Infect. Dis.* 20:223. 10.1186/s12879-020-4943-x 32171281 PMC7071708

[B54] Jones B. A. Grace D. Kock R. Alonso S. Rushton J. Said M. Y. (2013). Zoonosis emergence linked to agricultural intensification and environmental change. *Proc. Natl. Acad. Sci. USA* 110 8399–8404. 10.1073/pnas.1208059110 23671097 PMC3666729

[B55] Kabir M. H. B. Itoh M. Shehata A. A. Bando H. Fukuda Y. Murakoshi F. (2020). Distribution of *Cryptosporidium* species isolated from diarrhoeic calves in Japan. *Parasitol. Int.* 78:102153. 10.1016/j.parint.2020.102153 32504804

[B56] Karmali M. A. (2018). Factors in the emergence of serious human infections associated with highly pathogenic strains of shiga toxin-producing *Escherichia coli*. *Int. J. Med. Microbiol.* 308 1067–1072. 10.1016/j.ijmm.2018.08.005 30146439

[B57] Kawasaki J. Kojima S. Tomonaga K. Horie M. (2021). Hidden viral sequences in public sequencing data and warning for future emerging diseases. *mBio* 12:e0163821. 10.1128/mBio.01638-21 34399612 PMC8406186

[B58] Kerdsin A. Takeuchi D. Nuangmek A. Akeda Y. Gottschalk M. Oishi K. (2020). Genotypic comparison between *Streptococcus suis* isolated from pigs and humans in Thailand. *Pathogens* 9:50. 10.3390/pathogens9010050 31936553 PMC7168618

[B59] Kinross P. Beser J. Troell K. Axen C. Bjorkman C. Lebbad M. (2015). *Cryptosporidium parvum* infections in a cohort of veterinary students in Sweden. *Epidemiol. Infect.* 143 2748–2756. 10.1017/S0950268814003318 25633822 PMC4594045

[B60] Kinross P. Petersen A. Skov R. Van Hauwermeiren E. Pantosti A. Laurent F. (2017). Livestock-associated meticillin-resistant *Staphylococcus aureus* (MRSA) among human MRSA isolates, European Union/European Economic Area countries, 2013. *Euro. Surveill.* 22 16–96. 10.2807/1560-7917.ES.2017.22.44.16-00696 29113628 PMC5710135

[B61] Kirkhorn S. R. (2002). Community and environmental health effects of concentrated animal feeding operations. *Minn. Med.* 85 38–43. 12416314

[B62] Kittl S. Brodard I. Heim D. Andina-Pfister P. Overesch G. (2020). Methicillin-resistant *Staphylococcus aureus* strains in Swiss pigs and their relation to isolates from farmers and veterinarians. *Appl. Environ. Microbiol.* 86 e1819–e1865. 10.1128/AEM.01865-19 31836575 PMC7028968

[B63] Klumb C. A. Scheftel J. M. Smith K. E. (2020). Animal agriculture exposures among Minnesota residents with zoonotic enteric infections, 2012-2016. *Epidemiol. Infect.* 148:e55. 10.1017/S0950268819002309 32172700 PMC7078579

[B64] Kock R. Ballhausen B. Bischoff M. Cuny C. Eckmanns T. Fetsch A. (2014). The impact of zoonotic MRSA colonization and infection in Germany. *Berl Munch Tierarztl Wochenschr* 127 384–398. 25868166

[B65] Koneswaran G. Nierenberg D. (2008). Global farm animal production and global warming: impacting and mitigating climate change. *Environ. Health Perspect.* 116 578–582. 10.1289/ehp.11034 18470284 PMC2367646

[B66] Koutsoumanis K. Allende A. Alvarez-Ordonez A. Bolton D. Bover-Cid S. Chemaly M. (2019). *Salmonella* control in poultry flocks and its public health impact. *EFSA J.* 17:e05596. 10.2903/j.efsa.2019.5596 32626222 PMC7009056

[B67] La Rosa G. Della Libera S. Brambilla M. Bisaglia C. Pisani G. Ciccaglione A. R. (2017). Hepatitis E Virus (Genotype 3) in slurry samples from swine farming activities in Italy. *Food Eviron. Virol.* 9 219–229. 10.1007/s12560-016-9270-4 27853931

[B68] Larsen J. Petersen A. Larsen A. R. Sieber R. N. Stegger M. Koch A. (2017). Emergence of livestock-associated methicillin-resistant *Staphylococcus aureus* bloodstream infections in Denmark. *Clin. Infect. Dis.* 65 1072–1076. 10.1093/cid/cix504 28575216 PMC5850567

[B69] Lebbad M. Winiecka-Krusnell J. Stensvold C. R. Beser J. (2021). High diversity of *Cryptosporidium* species and subtypes identified in cryptosporidiosis acquired in Sweden and abroad. *Pathogens* 10:523. 10.3390/pathogens10050523 33926039 PMC8147002

[B70] Lee G. Y. Seong H. J. Sul W. J. Yang S. J. (2021). Genomic information on linezolid-resistant sequence-type 398 livestock-associated methicillin-resistant *Staphylococcus aureus* isolated from a pig. *Foodborne Pathog. Dis.* 18 378–387. 10.1089/fpd.2020.2882 33656917

[B71] Levallois P. Chevalier P. Gingras S. Dery P. Payment P. Michel P. (2014). Risk of infectious gastroenteritis in young children living in Quebec rural areas with intensive animal farming: results of a case-control study (2004-2007). *Zoonoses Public Health* 61 28–38. 10.1111/zph.12039 23406420 PMC7165781

[B72] Li H. Wu J. Sheng Y. Lu Q. Liu B. Chen Y. (2019). Prevalence of hepatitis E virus (HEV) infection in various pig farms from shaanxi province, china: first detection of HEV RNA in pig semen. *Transbound Emerg. Dse.* 66 72–82. 10.1111/tbed.12966 30043495

[B73] Lienen T. Schnitt A. Cuny C. Maurischat S. Tenhagen B. A. (2021). Phylogenetic tracking of LA-MRSA ST398 intra-farm transmission among animals, humans and the environment on German dairy farms. *Microorganisms* 9:1119. 10.3390/microorganisms9061119 34064246 PMC8224388

[B74] Ligda P. Claerebout E. Kostopoulou D. Zdragas A. Casaert S. Robertson L. J. (2020). *Cryptosporidium* and *Giardia* in surface water and drinking water: Animal sources and towards the use of a machine-learning approach as a tool for predicting contamination. *Environ. Poll.* 264:114766. 10.1016/j.envpol.2020.114766 32417583

[B75] Liu W. J. Xiao H. Dai L. Liu D. Chen J. Qi X. (2021). Avian influenza A (H7N9) virus: from low pathogenic to highly pathogenic. *Front. Med.* 15:507–527. 10.1007/s11684-020-0814-5 33860875 PMC8190734

[B76] Loeck B. K. Pedati C. Iwen P. C. McCutchen E. Roellig D. M. Hlavsa M. C. (2020). Genotyping and subtyping *Cryptosporidium* to identify risk factors and transmission patterns - Nebraska, 2015-2017. *Morb. Mortal. Wkly. Rep.* 69 335–338. 10.15585/mmwr.mm6912a4 32214081 PMC7725511

[B77] Lombardelli J. A. Tomazic M. L. Schnittger L. Tiranti K. I. (2019). Prevalence of *Cryptosporidium parvum* in dairy calves and GP60 subtyping of diarrheic calves in central Argentina. *Parasitol. Res.* 118 2079–2086. 10.1007/s00436-019-06366-y 31187226 PMC7087732

[B78] Lu L. Sikkema R. S. Velkers F. C. Nieuwenhuijse D. F. Fischer E. A. J. Meijer P. A. (2021). Adaptation, spread and transmission of SARS-CoV-2 in farmed minks and associated humans in the Netherlands. *Nat. Commun.* 12:6802. 10.1038/s41467-021-27096-9 34815406 PMC8611045

[B79] Luiken R. E. C. Van Gompel L. Munk P. Sarrazin S. Joosten P. Dorado-Garcia A. (2019). Associations between antimicrobial use and the faecal resistome on broiler farms from nine European countries. *J. Antimicrob. Chemother.* 74 2596–2604. 10.1093/jac/dkz235 31199864 PMC6916135

[B80] Maikai B. V. Umoh J. U. Kwaga J. K. Lawal I. A. Maikai V. A. Cama V. (2011). Molecular characterization of *Cryptosporidium* spp. in native breeds of cattle in Kaduna State. *Nigeria. Vet. Parasitol.* 178 241–245. 10.1016/j.vetpar.2010.12.048 21277091

[B81] Majeed Q. A. H. El-Azazy O. M. E. Abdou N. M. I. Al-Aal Z. A. El-Kabbany A. I. Tahrani L. M. A. (2018). Epidemiological observations on cryptosporidiosis and molecular characterization of *Cryptosporidium* spp. in sheep and goats in Kuwait. *Parasitol. Res.* 117 1631–1636. 10.1007/s00436-018-5847-1 29594423

[B82] Martins T. A. Seixas M. Brito D. R. B. Martins F. D. C. Cardim S. T. Melo P. (2018). First identification of *Cryptosporidium parvum* subtype IIaA20G1R1 in water buffalos (*Bubalus bubalis*). *Res. Vet. Sci.* 118 181–183. 10.1016/j.rvsc.2018.02.002 29514125

[B83] Marus J. R. Magee M. J. Manikonda K. Nichols M. C. (2019). Outbreaks of *Salmonella enterica* infections linked to animal contact: Demographic and outbreak characteristics and comparison to foodborne outbreaks-United States, 2009-2014. *Zoonoses Public Health* 66 370–376. 10.1111/zph.12569 30821071 PMC6779119

[B84] Money P. Kelly A. F. Gould S. W. Denholm-Price J. Threlfall E. J. Fielder M. D. (2010). Cattle, weather and water: mapping *Escherichia coli* O157:H7 infections in humans in England and Scotland. *Environ. Microbiol.* 12 2633–2644. 10.1111/j.1462-2920.2010.02293.x 20642796

[B85] Moyer M. W. (2016). The Looming threat of factory superbugs. *Sci. Am.* 315 70–79. 10.1038/scientificamerican1216-70 28004688

[B86] Mphephu M. G. Ekwanzala M. D. Momba M. N. B. (2021). *Cryptosporidium* species and subtypes in river water and riverbed sediment using next-generation sequencing. *Int. J. Parasitol.* 51 339–351. 10.1016/j.ijpara.2020.10.005 33421439

[B87] Nakaminami H. Kawasaki H. Takadama S. Kaneko H. Suzuki Y. Maruyama H. (2021). Possible dissemination of a Panton-Valentine leukocidin-positive livestock-associated methicillin-resistant *Staphylococcus aureus* CC398 clone in Tokyo. Japan. *Jpn. J. Infect. Dis.* 74 82–84. 10.7883/yoken.JJID.2020.345 32741933

[B88] NASPHV (2011). Compendium of measures to prevent disease associated with animals in public settings, 2011: national association of state public health veterinarians, inc. *MMWR Recomm. Rep.* 60 1–24. 21546893

[B89] Nguyen S. T. Fukuda Y. Tada C. Sato R. Duong B. Nguyen D. T. (2012). Molecular characterization of *Cryptosporidium* in native beef calves in central Vietnam. *Parasitol. Res.* 111 1817–1820. 10.1007/s00436-012-3038-z 22828931

[B90] Nic Lochlainn L. M. Sane J. Schimmer B. Mooij S. Roelfsema J. van Pelt W. (2019). Risk factors for sporadic cryptosporidiosis in the Netherlands: analysis of a 3-year population based case-control study coupled with genotyping, 2013-2016. *J. Infect. Dis.* 219 1121–1129. 10.1093/infdis/jiy634 30395258 PMC6420163

[B91] Nicholson C. W. Campagnolo E. R. Boktor S. W. Butler C. L. (2020). Zoonotic disease awareness survey of backyard poultry and swine owners in southcentral Pennsylvania. *Zoonoses Public Health* 67 280–290. 10.1111/zph.12686 32020787 PMC7231624

[B92] Nunez I. A. Ross T. M. (2019). A review of H5Nx avian influenza viruses. *Ther. Adv. Vacc. Immunother.* 7 1–15. 10.1177/2515135518821625 30834359 PMC6391539

[B93] O’Leary J. K. Blake L. Corcoran G. D. Sleator R. D. Lucey B. (2020). Increased diversity and novel subtypes among clinical *Cryptosporidium parvum* and *Cryptosporidium hominis* isolates in Southern Ireland. *Exp. Parasitol.* 218:107967. 10.1016/j.exppara.2020.107967 32858044

[B94] Pallerla S. R. Harms D. Johne R. Todt D. Steinmann E. Schemmerer M. (2020). Hepatitis E virus infection: circulation, molecular epidemiology, and impact on global health. *Pathogens* 9:856. 10.3390/pathogens9100856 33092306 PMC7589794

[B95] Palomares Velosa J. E. Salman M. D. Roman-Muniz I. N. Reynolds S. Linke L. Magnuson R. (2020). Socio-ecological factors of zoonotic diseases exposure in Colorado dairy workers. *J. Agromed.* 26 151–161. 10.1080/1059924X.2020.1725700 32052708 PMC9552966

[B96] Philippon D. A. M. Wu P. Cowling B. J. Lau E. H. Y. (2020). Avian influenza human infections at the human-animal interface. *J. Infect. Dis.* 222 528–537. 10.1093/infdis/jiaa105 32157291

[B97] Pirolo M. Sieber R. N. Moodley A. Visaggio D. Artuso I. Gioffre A. (2020). Local and transboundary transmissions of methicillin-resistant *Staphylococcus aureus* sequence type 398 through pig trading. *Appl. Environ. Microbiol.* 86 e420–e430. 10.1128/AEM.00430-20 32358001 PMC7301864

[B98] Pirolo M. Visaggio D. Gioffre A. Artuso I. Gherardi M. Pavia G. (2019). Unidirectional animal-to-human transmission of methicillin-resistant *Staphylococcus aureus* ST398 in pig farming; evidence from a surveillance study in southern Italy. *Antimicrob. Resist. Infect. Control* 8:187. 10.1186/s13756-019-0650-z 31832187 PMC6873530

[B99] Pogreba-Brown K. O’Connor P. Matthews J. Barrett E. Bell M. L. (2018). Case-case analysis of *Campylobacter* and *Salmonella* - using surveillance data for outbreak investigations and monitoring routine risk factors. *Epidemiol. Infect.* 146 1916–1921. 10.1017/S0950268818002200 30092849 PMC6452982

[B100] Pohjola L. Nykasenoja S. Kivisto R. Soveri T. Huovilainen A. Hanninen M. L. (2016). Zoonotic public health hazards in backyard chickens. *Zoonoses Public Health* 63 420–430. 10.1111/zph.12247 26752227

[B101] Quan C. Wang Q. Zhang J. Zhao M. Dai Q. Huang T. (2019). Avian influenza A viruses among occupationally exposed populations, China, 2014-2016. *Emerg. Infect. Dis.* 25 2215–2225. 10.3201/eid2512.190261 31742536 PMC6874249

[B102] Quilez J. Torres E. Chalmers R. M. Hadfield S. J. Del Cacho E. Sanchez-Acedo C. (2008). *Cryptosporidium* genotypes and subtypes in lambs and goat kids in Spain. *Appl. Environ. Microbiol.* 74 6026–6031. 10.1128/AEM.00606-08 18621872 PMC2565967

[B103] Radon K. Schulze A. Ehrenstein V. van Strien R. T. Praml G. Nowak D. (2007). Environmental exposure to confined animal feeding operations and respiratory health of neighboring residents. *Epidemiology* 18 300–308. 10.1097/01.ede.0000259966.62137.84 17435437

[B104] Razakandrainibe R. Diawara E. H. I. Costa D. Le Goff L. Lemeteil D. Ballet J. J. (2018). Common occurrence of *Cryptosporidium hominis* in asymptomatic and symptomatic calves in France. *PLoS Negl. Trop. Dis.* 12:e0006355. 10.1371/journal.pntd.0006355 29596411 PMC5892941

[B105] Ren M. Wu F. Wang D. Li L. Y. Chang J. J. Lin Q. (2019). Molecular typing of *Cryptosporidium* species identified in fecal samples of yaks (*Bos grunniens*) of Qinghai Province. *China J. Parasitol.* 105 195–198. 10.1645/18-62 30835169

[B106] Rieux A. Chartier C. Pors I. Paraud C. (2013). Dynamics of excretion and molecular characterization of *Cryptosporidium* isolates in pre-weaned French beef calves. *Vet. Parasitol.* 195 169–172. 10.1016/j.vetpar.2012.12.043 23312870

[B107] Rohr J. R. Barrett C. B. Civitello D. J. Craft M. E. Delius B. DeLeo G. A. (2019). Emerging human infectious diseases and the links to global food production. *Nat. Sustain.* 2 445–456. 10.1038/s41893-019-0293-3 32219187 PMC7091874

[B108] Rosov K. A. Mallin M. A. Cahoon L. B. (2020). Waste nutrients from U.S. animal feeding operations: Regulations are inconsistent across states and inadequately assess nutrient export risk. *J. Environ. Manage.* 269:110738. 10.1016/j.jenvman.2020.110738 32560983

[B109] Rui P. Zhao F. Yan S. Wang C. Fu Q. Hao J. (2020). Detection of hepatitis E virus genotypes 3 and 4 in donkeys in northern China. *Equine. Vet. J.* 52 415–419. 10.1111/evj.13203 31746470

[B110] Russell S. Power M. Ens E. (2020). *Cryptosporidium* and *Giardia* in feral water buffalo (*Bubalus bubalis*) in the south east arnhem land indigenous protected area. *Australia Parasitol. Res.* 119 2149–2157. 10.1007/s00436-020-06703-6 32424553

[B111] Saeedi P. Yazdanparast M. Behzadi E. Salmanian A. H. Mousavi S. L. Nazarian S. (2017). A review on strategies for decreasing E. coli O157:H7 risk in animals. *Microb. Pathog.* 103 186–195. 10.1016/j.micpath.2017.01.001 28062285

[B112] Saenz R. A. Hethcote H. W. Gray G. C. (2006). Confined animal feeding operations as amplifiers of influenza. *Vector Borne Zoonotic. Dis.* 6 338–346. 10.1089/vbz.2006.6.338 17187567 PMC2042988

[B113] Sahraoui L. Thomas M. Chevillot A. Mammeri M. Polack B. Vallee I. (2019). Molecular characterization of zoonotic *Cryptosporidium* spp. and *Giardia duodenalis* pathogens in Algerian sheep. *Vet. Parasitol. Reg. Stud. Rep.* 16:100280. 10.1016/j.vprsr.2019.100280 31027593

[B114] Salaheen S. Chowdhury N. Hanning I. Biswas D. (2015). Zoonotic bacterial pathogens and mixed crop-livestock farming. *Poult. Sci.* 94 1398–1410. 10.3382/ps/peu055 25589077

[B115] Santin M. (2020). *Cryptosporidium* and *Giardia* in ruminants. *Vet. Clin. North Am. Food Anim. Pract.* 36 223–238. 10.1016/j.cvfa.2019.11.005 32029186

[B116] Santoro A. Dorbek-Kolin E. Jeremejeva J. Tummeleht L. Orro T. Jokelainen P. (2019). Molecular epidemiology of *Cryptosporidium* spp. in calves in Estonia: high prevalence of *Cryptosporidium parvum* shedding and 10 subtypes identified. *Parasitology* 146 261–267. 10.1017/S0031182018001348 30086806

[B117] Sasaki Y. Sakurada H. Yamanaka M. Nara K. Tanaka S. Uema M. (2021). Effectiveness of ear skin swabs for monitoring methicillin-resistant *Staphylococcus aureus* ST398 in pigs at abattoirs. *J. Vet. Med. Sci.* 83 112–115. 10.1292/jvms.20-0592 33431727 PMC7870411

[B118] Seman M. Gregova G. Korim P. (2020). Comparison of *Campylobacter* spp. and flock health indicators of broilers in Iceland. *Ann. Agric. Environ. Med.* 27 579–584. 10.26444/aaem/127181 33356064

[B119] Shi J. Wen Z. Zhong G. Yang H. Wang C. Huang B. (2020). Susceptibility of ferrets, cats, dogs, and other domesticated animals to SARS-coronavirus 2. *Science* 368 1016–1020. 10.1126/science.abb7015 32269068 PMC7164390

[B120] Shu Y. Chen Y. Zhou S. Zhang S. Wan Q. Zhu C. (2019). Cross-sectional seroprevalence and genotype of hepatitis E virus in humans and swine in a high-density pig-farming area in central China. *Virol. Sin.* 34 367–376. 10.1007/s12250-019-00136-x 31264049 PMC6687807

[B121] Shwab E. K. Saraf P. Zhu X. Q. Zhou D. H. McFerrin B. M. Ajzenberg D. (2018). Human impact on the diversity and virulence of the ubiquitous zoonotic parasite *Toxoplasma gondii*. *Proc. Natl. Acad. Sci. USA* 115 E6956–E6963. 10.1073/pnas.1722202115 29967142 PMC6055184

[B122] Sieber R. N. Larsen A. R. Urth T. R. Iversen S. Moller C. H. Skov R. L. (2019). Genome investigations show host adaptation and transmission of LA-MRSA CC398 from pigs into Danish healthcare institutions. *Sci. Rep.* 9:18655. 10.1038/s41598-019-55086-x 31819134 PMC6901509

[B123] Sieber R. N. Skov R. L. Nielsen J. Schulz J. Price L. B. Aarestrup F. M. (2018). Drivers and dynamics of methicillin-resistant livestock-associated *Staphylococcus aureus* CC398 in pigs and humans in Denmark. *mBio* 9 e2118–e2142. 10.1128/mBio.02142-18 30425152 PMC6234867

[B124] Silverlas C. Blanco-Penedo I. (2013). *Cryptosporidium* spp. in calves and cows from organic and conventional dairy herds. *Epidemiol. Infect.* 141 529–539. 10.1017/S0950268812000830 22564291 PMC9196992

[B125] Silverlas C. Bosaeus-Reineck H. Naslund K. Bjorkman C. (2013). Is there a need for improved *Cryptosporidium* diagnostics in Swedish calves? *Int. J. Parasitol.* 43 155–161. 10.1016/j.ijpara.2012.10.009 23142404 PMC7094644

[B126] Sooryanarain H. Meng X. J. (2020). Swine hepatitis E virus: Cross-species infection, pork safety and chronic infection. *Virus Res.* 284:197985. 10.1016/j.virusres.2020.197985 32333941 PMC7249539

[B127] Sridhar S. Lo S. K. Xing F. Yang J. Ye H. Chan J. F. (2017). Clinical characteristics and molecular epidemiology of hepatitis E in Shenzhen. China: a shift toward foodborne transmission of hepatitis E virus infection. *Emerg. Microbes. Infect.* 6:e115. 10.1038/emi.2017.107 29259325 PMC5750461

[B128] Su C. P. Stover D. T. Buss B. F. Carlson A. V. Luckhaupt S. E. (2017). Occupational animal exposure among persons with campylobacteriosis and cryptosporidiosis - Nebraska, 2005-2015. *Morb. Mortal. Wkly. Rep.* 66 955–958. 10.15585/mmwr.mm6636a4 28910275 PMC5657915

[B129] Susilawathi N. M. Tarini N. M. A. Fatmawati N. N. D. Mayura P. I. B. Suryapraba A. A. A. Subrata M. (2019). *Streptococcus suis*-associated meningitis. Bali, Indonesia, 2014-2017. *Emerg. Infect. Dis.* 25 2235–2242. 10.3201/eid2512.181709 31742523 PMC6874276

[B130] Swaffer B. Abbott H. King B. van der Linden L. Monis P. (2018). Understanding human infectious *Cryptosporidium* risk in drinking water supply catchments. *Water Res.* 138 282–292. 10.1016/j.watres.2018.03.063 29614456

[B131] Symeonidou I. Tassis P. Gelasakis A. Tzika E. D. Papadopoulos E. (2020). Prevalence and risk factors of intestinal parasite infections in Greek swine farrow-to-finish farms. *Pathogens* 9:556. 10.3390/pathogens9070556 32664245 PMC7399844

[B132] Tack D. M. Ray L. Griffin P. M. Cieslak P. R. Dunn J. Rissman T. (2020). Preliminary incidence and trends of infections with pathogens transmitted commonly through food - foodborne diseases active surveillance network, 10 U.S. Sites, 2016-2019. *Morb. Mortal. Wkly. Rep.* 69 509–514. 10.15585/mmwr.mm6917a1 32352955 PMC7206985

[B133] Teixeira J. Mesquita J. R. Pereira S. S. Oliveira R. M. Abreu-Silva J. Rodrigues A. (2017). Prevalence of hepatitis E virus antibodies in workers occupationally exposed to swine in Portugal. *Med. Microbiol. Immunol.* 206 77–81. 10.1007/s00430-016-0484-8 27770276

[B134] Thomas-Lopez D. Muller L. Vestergaard L. S. Christoffersen M. Andersen A. M. Jokelainen P. (2020). Veterinary students have a higher risk of contracting cryptosporidiosis when calves with high fecal *Cryptosporidium* loads are used for fetotomy exercises. *Appl. Environ. Microbiol.* 86 e1220–e1250. 10.1128/AEM.01250-20 32709724 PMC7499042

[B135] Thomson S. Innes E. A. Jonsson N. N. Katzer F. (2019). Shedding of *Cryptosporidium* in calves and dams: evidence of re-infection and shedding of different gp60 subtypes. *Parasitology* 146 1404–1413. 10.1017/S0031182019000829 31327324

[B136] Wang M. Du P. Wang J. Lan R. Huang J. Luo M. (2019). Genomic epidemiology of *Streptococcus suis* sequence type 7 sporadic infections in the Guangxi Zhuang Autonomous Region of China. *Pathogens* 8:187. 10.3390/pathogens8040187 31614790 PMC6963630

[B137] Wu Y. Chen Y. Chang Y. Zhang X. Li D. Wang L. (2020). Genotyping and identification of *Cryptosporidium* spp., *Giardia duodenalis* and *Enterocytozoon bieneusi* from free-range Tibetan yellow cattle and cattle-yak in Tibet, China. *Acta Trop.* 212:105671. 10.1016/j.actatropica.2020.105671 32822671

[B138] Xiao L. (2010). Molecular epidemiology of cryptosporidiosis: an update. *Exp. Parasitol.* 124 80–89. 10.1016/j.exppara.2009.03.018 19358845

[B139] Xiao L. Feng Y. (2017). Molecular epidemiologic tools for waterborne pathogens *Cryptosporidium* spp. and *Giardia duodenalis*. *Food Waterborne Parasitol.* 9 14–32. 10.1016/j.fawpar.2017.09.002 32095639 PMC7034008

[B140] Yang X. Guo Y. Xiao L. Feng Y. (2021). Molecular epidemiology of human cryptosporidiosis in low- and middle-income countries. *Clin. Microbiol. Rev.* 34 e19–e87. 10.1128/CMR.00087-19 33627442 PMC8549823

[B141] Yu F. Cienfuegos-Gallet A. V. Cunningham M. H. Jin Y. Wang B. Kreiswirth B. N. (2021). Molecular evolution and adaptation of livestock-associated methicillin-resistant *Staphylococcus aureus* (LA-MRSA) sequence type 9. *mSystems* 6:e0049221. 10.1128/mSystems.00492-21 34156294 PMC8269235

[B142] Yue N. Wang Q. Zheng M. Wang D. Duan C. Yu X. (2019). Prevalence of hepatitis E virus infection among people and swine in mainland China: a systematic review and meta-analysis. *Zoonoses. Public Health* 66 265–275. 10.1111/zph.12555 30884147

[B143] Zahedi A. Ryan U. (2020). *Cryptosporidium* - An update with an emphasis on foodborne and waterborne transmission. *Res. Vet. Sci.* 132 500–512. 10.1016/j.rvsc.2020.08.002 32805698

[B144] Zahedi A. Ryan U. Rawlings V. Greay T. Hancock S. Bruce M. (2020). *Cryptosporidium* and *Giardia* in dam water on sheep farms - an important source of transmission? *Vet. Parasitol.* 288:109281. 10.1016/j.vetpar.2020.109281 33142151

[B145] Zhou J. H. Shang Y. Cao X. A. Wang Y. N. Liu Y. Hu Y. (2019). Potential effects of hepatitis E virus infection in swine on public health in China. *Infect. Genet. Evol.* 68 113–118. 10.1016/j.meegid.2018.12.017 30562577

