# Supplementary material for: Association of Common Zoonotic Pathogens With Concentrated Animal Feeding Operations
Source: Front Microbiol. 2022 Jan 10;12:810142. doi: 10.3389/fmicb.2021.810142 (PMC8784678; doi:10.3389/fmicb.2021.810142)
Supplement: Supplementary file 1 [file Data_Sheet_1.docx]

**Supplementary Table 1.** Occurrence of *Cryptosporidium parvum* and *C. hominis* in human samples in industrialized nations in reports during 2010-2021*

| Study area | *Cryptosporidium* cases | *C. hominis* | *C. parvum* | Others | Reference |
| --- | --- | --- | --- | --- | --- |
| UK | 14026 | 7490 | 6423 | 164 | (Elwin et al., 2012) |
|  | 4379 | 2516 | 1693 | 62 | (Chalmers et al., 2011a) |
|  | 445 | 187 | 256 | 2 | (Deshpande et al., 2015) |
|  | 565 | 276 | 284 | 5 | (Pollock et al., 2010) |
| Ireland | 163 | 22 | 141 | 0 | (O'Leary et al., 2020a; O'Leary et al., 2020b) |
| France | 310 | 75 | 222 | 13 | (Costa et al., 2020) |
|  | 87 | 19 | 56 | 12 | (Costa et al., 2018) |
|  | 310 | 113 | 168 | 29 | (Network, 2010) |
| Spain | 81 | 66 | 15 | 0 | (Azcona-Gutierrez et al., 2017) |
|  | 69 | 61 | 7 | 1 | (Segura et al., 2015) |
|  | 44 | 41 | 3 | 0 | (Ramo et al., 2015) |
|  | 486 | 318 | 164 | 4 | (Abal-Fabeiro et al., 2015) |
|  | 550 | 370 | 176 | 4 | (Abal-Fabeiro et al., 2014) |
|  | 72 | 30 | 40 | 2 | (Navarro et al., 2013) |
|  | 61 | 27 | 31 | 0 | (Martin-Ampudia et al., 2012) |
| Italy | 9 | 1 | 8 | 0 | (Del Chierico et al., 2011) |
| Sweden | 398 | 50 | 300 | 49 | (Lebbad et al., 2021) |
|  | 195 | 66 | 112 | 17 | (Insulander et al., 2013) |
| Denmark | 43 | 8 | 34 | 1 | (Stensvold et al., 2015) |
| Netherlands | 427 | 123 | 304 | 0 | (Nic Lochlainn et al., 2019) |
| Slovakia | 20 | 3 | 17 | 0 | (Hatalova et al., 2018) |
|  | 5 | 3 | 1 | 1 | (Petrincova et al., 2015) |
| Romania | 4 | 0 | 4 | 0 | (Vieira et al., 2015) |
| Europe subtotal | 22749 | 11865 | 10459 | 366 | - |
| Qatar | 38 | 5 | 37 | 3 | (Boughattas et al., 2019) |
|  | 90 | 5 | 85 | 2 | (Boughattas et al., 2017) |
| Iran | 27 | 7 | 19 | 1 | (Ghafari et al., 2018) |
|  | 17 | 0 | 17 | 0 | (Kiani et al., 2017) |
|  | 5 | 0 | 5 | 0 | (Berahmat et al., 2017) |
|  | 7 | 0 | 7 | 0 | (Ranjbar et al., 2016) |
|  | 15 | 0 | 15 | 0 | (Sharbatkhori et al., 2015) |
|  | 2 | 0 | 2 | 0 | (Mahdavi Poor et al., 2015) |
|  | 16 | 4 | 11 | 1 | (Rafiei et al., 2014) |
|  | 5 | 2 | 2 | 1 | (Ghaffari and Kalantari, 2014) |
|  | 11 | 4 | 7 | 0 | (Izadi et al., 2012) |
|  | 19 | 2 | 17 |  | (Taghipour et al., 2011) |
|  | 25 | 3 | 22 | 0 | (Nazemalhosseini-Mojarad et al., 2011) |
| Kuwait | 83 | 22 | 61 | 0 | (Iqbal et al., 2011) |
| Jordan | 44 | 20 | 22 | 2 | (Hijjawi et al., 2010) |
|  | 32 | 0 | 32 | 0 | (Hijjawi et al., 2017) |
|  | 4 | 2 | 2 | 0 | (Hijjawi et al., 2016) |
| Lebanon | 15 | 10 | 5 | 0 | (Osman et al., 2015) |
| Yemen | 33 | 1 | 32 | 0 | (Alyousefi et al., 2013) |
| S. Korea | 32 | 0 | 32 | 0 | (Ma et al., 2019) |
| Indonesia | 28 | 23 | 0 | 5 | (Kurniawan et al., 2013) |
| Malaysia | 9 | 0 | 9 | 0 | (Sahimin et al., 2018) |
|  | 32 | 2 | 27 | 3 | (Asma et al., 2015) |
|  | 18 | 5 | 13 | 0 | (Iqbal et al., 2012) |
|  | 25 | 6 | 16 | 3 | (Lim et al., 2011) |
| Australia | 102 | 98 | 1 | 3 | (Braima et al., 2021) |
|  | 109 | 94 | 12 | 3 | (Braima et al., 2019) |
|  | 324 | 259 | 54 | 11 | (Ng-Hublin et al., 2017) |
|  | 5 | 2 | 1 | 2 | (Ebner et al., 2015) |
|  | 82 | 66 | 15 | 1 | (Koehler et al., 2014) |
|  | 94 | 71 | 21 | 2 | (Koehler et al., 2013) |
|  | 261 | 172 | 87 | 2 | (Waldron et al., 2011) |
|  | 248 | 195 | 49 | 4 | (Ng et al., 2010) |
| New Zealand | 2598 | 1083 | 1462 | 53 | (Garcia et al., 2020) |
|  | 534 | 241 | 287 | 6 | (Garcia et al., 2017) |
| Asia and Oceania subtotal | 4989 | 2404 | 2486 | 108 | - |
| United States | 40 | 9 | 24 | 3 | (Mergen et al., 2020) |
|  | 149 | 80 | 58 | 11 | (Loeck et al., 2020) |
| Canada | 129 | 28 | 95 | 10 | (Guy et al., 2021) |
|  | 47 | 11 | 35 | 1 | (Ayres Hutter et al., 2020) |
|  | 15 | 0 | 15 | 0 | (Iqbal et al., 2015) |
|  | 25 | 9 | 16 | 0 | (Budu-Amoako et al., 2012) |
| Mexico | 32 | 13 | 19 | 0 | (Urrea-Quezada et al., 2018) |
|  | 12 | 10 | 2 | 0 | (Valenzuela et al., 2014) |
| Colombia | 15 | 2 | 4 | 9 | (Higuera et al., 2020) |
|  | 7 | 6 | 0 | 1 | (Galvan-Diaz et al., 2020) |
|  | 4 | 1 | 3 | 0 | (Villamizar et al., 2019) |
|  | 5 | 2 | 2 | 1 | (Sanchez et al., 2017) |
| Guatemala | 4 | 2 | 2 | 0 | (Velasquez et al., 2011) |
| Chile | 29 | 11 | 16 | 2 | (Neira et al., 2012) |
| Brazil | 19 | 15 | 5 | 0 | (Peralta et al., 2016) |
|  | 42 | 37 | 5 | 0 | (Rolando et al., 2012) |
| Argentina | 15 | 10 | 6 | 0 | (Peralta et al., 2016) |
|  | 3 | 3 | 2 | 0 | (Netor Velasquez et al., 2012) |
| America subtotal | 592 | 249 | 309 | 38 | - |
| **Total** | **28330** | **14518** | **13254** | **512** | **-** |

*See previous reviews for summaries of data from studies before 2010 (Xiao, 2010) and those from low- and middle-income countries (Yang et al., 2021).

**Supplementary Table 2.** Occurrence of major *Cryptosporidium parvum* subtype families in human samples in industrialized nations in reports during 2010-2021*

| Area | *C. parvum* cases | IIa | IId | IIc | Other | Reference |
| --- | --- | --- | --- | --- | --- | --- |
| Sweden | 299 | 164 | 118 | 2 | 15 | (Lebbad et al., 2021) |
|  | 107 | 69 | 24 | 11 | 3 | (Insulander et al., 2013) |
| Denmark | 15 | 15 | 0 | 0 | 0 | (Stensvold et al., 2015) |
| UK | 87 | 82 | 2 | 2 | 1 | (Deshpande et al., 2015) |
|  | 66 | 56 | 9 | 1 |  | (Chalmers et al., 2011b) |
| Ireland | 129 | 129 | 0 | 0 | 0 | (O'Leary et al., 2020b) |
|  | 249 | 249 | 0 | 0 | 0 | (Zintl et al., 2011) |
| France | 222 | 147 | 58 | 13 | 4 | (Costa et al., 2020) |
| Italy | 8 | 4 | 0 | 4 | 0 | (Del Chierico et al., 2011) |
| Spain | 8 | 6 | 2 | 0 | 0 | (Azcona-Gutierrez et al., 2017) |
|  | 7 | 6 | 1 | 0 | 0 | (Segura et al., 2015) |
|  | 3 | 3 | 0 | 0 | 0 | (Ramo et al., 2015) |
|  | 164 | 146 | 3 | 0 | 15 | (Abal-Fabeiro et al., 2015) |
|  | 173 | 155 | 3 | 0 | 16 | (Abal-Fabeiro et al., 2014) |
| Slovakia | 17 | 17 | 0 | 0 | 0 | (Hatalova et al., 2018) |
|  | 1 | 1 | 0 | 0 | 0 | (Petrincova et al., 2015) |
| Romania | 4 | 0 | 4 | 0 | 0 | (Vieira et al., 2015) |
| Europe subtotal | 1559 | 1249 | 224 | 33 | 54 | - |
| Qatar | 31 | 0 | 31 | 0 | 0 | (Boughattas et al., 2019) |
|  | 31 | 0 | 31 | 0 | 0 | (Boughattas et al., 2017) |
| Iran | 17 | 6 | 11 | 0 | 0 | (Kiani et al., 2017) |
|  | 7 | 5 | 2 | 0 | 0 | (Ranjbar et al., 2016) |
|  | 15 | 7 | 8 | 0 | 0 | (Sharbatkhori et al., 2015) |
|  | 2 | 0 | 2 | 0 | 0 | (Ghaffari and Kalantari, 2014) |
|  | 17 | 6 | 11 | 0 | 0 | (Taghipour et al., 2011) |
|  | 22 | 8 | 14 | 0 | 0 | (Nazemalhosseini-Mojarad et al., 2011) |
| Kuwait | 61 | 29 | 20 | 12 | 0 | (Iqbal et al., 2011) |
| Jordan | 13 | 3 | 8 | 2 | 0 | (Hijjawi et al., 2010) |
|  | 2 | 1 | 1 | 0 | 0 | (Hijjawi et al., 2016) |
|  | 32 | 32 | 0 | 0 | 0 | (Hijjawi et al., 2017) |
| Lebanon | 5 | 5 | 0 | 0 | 0 | (Osman et al., 2015) |
| Yemen | 7 | 7 | 0 | 0 | 0 | (Alyousefi et al., 2013) |
| S. Korea | 11 | 11 | 0 | 0 | 0 | (Ma et al., 2019) |
| Malaysia | 13 | 12 | 1 | 0 | 0 | (Iqbal et al., 2012) |
|  | 1 | 0 | 1 | 0 | 0 | (Lim et al., 2011) |
| Australia | 12 | 9 | 0 | 3 | 0 | (Braima et al., 2019) |
|  | 53 | 52 | 1 | 0 | 0 | (Ng-Hublin et al., 2017) |
|  | 14 | 14 | 0 | 0 | 0 | (Koehler et al., 2014) |
|  | 21 | 21 | 0 | 0 | 0 | (Koehler et al., 2013) |
|  | 80 | 79 | 1 | 0 | 0 | (Waldron et al., 2011) |
|  | 49 | 48 | 1 | 0 | 0 | (Ng et al., 2010) |
| New Zealand | 1464 | 1105 | 343 | 9 | 5 | (Garcia et al., 2020) |
|  | 287 | 217 | 66 | 3 | 1 | (Garcia et al., 2017) |
| Asia subtotal | 2267 | 1677 | 553 | 29 | 6 | - |
| Canada | 94 | 86 | 4 | 4 | 0 | (Guy et al., 2021) |
|  | 24 | 24 | 0 | 0 | 0 | (Ayres Hutter et al., 2020) |
|  | 7 | 7 | 0 | 0 | 0 | (Iqbal et al., 2015) |
|  | 5 | 5 | 0 | 0 | 0 | (Budu-Amoako et al., 2012) |
| Mexico | 19 | 17 | 0 | 2 | 0 | (Urrea-Quezada et al., 2018) |
|  | 2 | 2 | 0 | 0 | 0 | (Valenzuela et al., 2014) |
| Colombia | 3 | 3 | 0 | 0 | 0 | (Villamizar et al., 2019) |
|  | 2 | 0 | 0 | 2 | 0 | (Sanchez et al., 2017) |
| Brazil | 1 | 0 | 0 | 1 | 0 | (Peralta et al., 2016) |
| Argentia | 2 | 2 | 0 | 0 | 0 | (Peralta et al., 2016) |
| America subtotal | 159 | 146 | 4 | 9 | 0 | - |
| **Total** | **3985** | **3072** | **781** | **71** | **60** | **-** |

*See previous reviews for summaries of data from studies before 2010 (Xiao, 2010) and those from low- and middle-income countries (Yang et al., 2021).

**References**

Abal-Fabeiro, J.L., Maside, X., Llovo, J., Bartolome, C. (2015) Aetiology and epidemiology of human cryptosporidiosis cases in Galicia (NW Spain), 2000-2008. *Epidemiol Infect* 143, 3022-3035.

Abal-Fabeiro, J.L., Maside, X., Llovo, J., Bello, X., Torres, M., Trevino, M., Moldes, L., Munoz, A., Carracedo, A., Bartolome, C. (2014) High-throughput genotyping assay for the large-scale genetic characterization of *Cryptosporidium* parasites from human and bovine samples. *Parasitology* 141, 491-500.

Alyousefi, N.A., Mahdy, M.A., Lim, Y.A., Xiao, L., Mahmud, R. (2013) First molecular characterization of *Cryptosporidium* in Yemen. *Parasitology* 140, 729-734.

Asma, I., Sim, B.L., Brent, R.D., Johari, S., Yvonne Lim, A.L. (2015) Molecular epidemiology of *Cryptosporidium* in HIV/AIDS patients in Malaysia. *Trop Biomed* 32, 310-322.

Ayres Hutter, J., Dion, R., Irace-Cima, A., Fiset, M., Guy, R., Dixon, B., Aguilar, J.L., Trepanier, J., Thivierge, K. (2020) *Cryptosporidium* spp.: Human incidence, molecular characterization and associated exposures in Quebec, Canada (2016-2017). *PLoS ONE* 15, e0228986.

Azcona-Gutierrez, J.M., de Lucio, A., Hernandez-de-Mingo, M., Garcia-Garcia, C., Soria-Blanco, L.M., Morales, L., Aguilera, M., Fuentes, I., Carmena, D. (2017) Molecular diversity and frequency of the diarrheagenic enteric protozoan *Giardia duodenalis* and *Cryptosporidium* spp. in a hospital setting in Northern Spain. *PLoS ONE* 12, e0178575.

Berahmat, R., Mahami-Oskouei, M., Rezamand, A., Spotin, A., Aminisani, N., Ghoyounchi, R., Madadi, S. (2017) *Cryptosporidium* infection in children with cancer undergoing chemotherapy: how important is the prevention of opportunistic parasitic infections in patients with malignancies? *Parasitol Res* 116, 2507-2515.

Boughattas, S., Behnke, J.M., Al-Ansari, K., Sharma, A., Abu-Alainin, W., Al-Thani, A., Abu-Madi, M.A. (2017) Molecular analysis of the enteric protozoa associated with acute diarrhea in hospitalized children. *Front Cell Infect Microbiol* 7, 343.

Boughattas, S., Behnke, J.M., Al-Sadeq, D., Ismail, A., Abu-Madi, M. (2019) *Cryptosporidium* spp., prevalence, molecular characterisation and socio-demographic risk factors among immigrants in Qatar. *PLoS Negl Trop Dis* 13, e0007750.

Braima, K., Zahedi, A., Egan, S., Austen, J., Xiao, L., Feng, Y., Witham, B., Pingault, N., Perera, S., Oskam, C., Reid, S., Ryan, U. (2021) Molecular analysis of cryptosporidiosis cases in Western Australia in 2019 and 2020 supports the occurrence of two swimming pool associated outbreaks and reveals the emergence of a rare *C. hominis* IbA12G3 subtype. *Infect Genet Evol* 92, 104859.

Braima, K., Zahedi, A., Oskam, C., Reid, S., Pingault, N., Xiao, L., Ryan, U. (2019) Retrospective analysis of *Cryptosporidium* species in Western Australian human populations (2015-2018), and emergence of the *C. hominis* IfA12G1R5 subtype. *Infect Genet Evol* 73, 306-313.

Budu-Amoako, E., Greenwood, S.J., Dixon, B.R., Sweet, L., Ang, L., Barkema, H.W., McClure, J.T. (2012) Molecular epidemiology of *Cryptosporidium* and *Giardia* in humans on prince edward island, Canada: evidence of zoonotic transmission from cattle. *ZoonosesPublic Health* 59, 424-433.

Chalmers, R.M., Smith, R., Elwin, K., Clifton-Hadley, F.A., Giles, M. (2011a) Epidemiology of anthroponotic and zoonotic human cryptosporidiosis in England and Wales, 2004-2006. *Epidemiol Infect* 139, 700-712.

Chalmers, R.M., Smith, R.P., Hadfield, S.J., Elwin, K., Giles, M. (2011b) Zoonotic linkage and variation in *Cryptosporidium parvum* from patients in the United Kingdom. *Parasitol Res* 108, 1321-1325.

Costa, D., Razakandrainibe, R., Sautour, M., Valot, S., Basmaciyan, L., Gargala, G., Lemeteil, D., French national network on surveillance of human, C., Favennec, L., Dalle, F. (2018) Human cryptosporidiosis in immunodeficient patients in France (2015-2017). *Exp Parasitol* 192, 108-112.

Costa, D., Razakandrainibe, R., Valot, S., Vannier, M., Sautour, M., Basmaciyan, L., Gargala, G., Viller, V., Lemeteil, D., Ballet, J.J., French National Network on Surveillance of Human, C., Dalle, F., Favennec, L. (2020) Epidemiology of cryptosporidiosis in France from 2017 to 2019. *Microorganisms* 8.

Del Chierico, F., Onori, M., Di Bella, S., Bordi, E., Petrosillo, N., Menichella, D., Caccio, S.M., Callea, F., Putignani, L. (2011) Cases of cryptosporidiosis co-infections in AIDS patients: a correlation between clinical presentation and GP60 subgenotype lineages from aged formalin-fixed stool samples. *Ann Trop Med Parasitol* 105, 339-349.

Deshpande, A.P., Jones, B.L., Connelly, L., Pollock, K.G., Brownlie, S., Alexander, C.L. (2015) Molecular characterization of *Cryptosporidium parvum* isolates from human cryptosporidiosis cases in Scotland. *Parasitology* 142, 318-325.

Ebner, J., Koehler, A.V., Robertson, G., Bradbury, R.S., Jex, A.R., Haydon, S.R., Stevens, M.A., Norton, R., Joachim, A., Gasser, R.B. (2015) Genetic analysis of *Giardia* and *Cryptosporidium* from people in Northern Australia using PCR-based tools. *Infect Genet Evol* 36, 389-395.

Elwin, K., Hadfield, S.J., Robinson, G., Chalmers, R.M. (2012) The epidemiology of sporadic human infections with unusual cryptosporidia detected during routine typing in England and Wales, 2000-2008. *Epidemiol Infect* 140, 673-683.

Galvan-Diaz, A.L., Bedoya-Urrego, K., Medina-Lozano, A., Uran-Velasquez, J., Alzate, J.F., Garcia-Montoya, G. (2020) Common occurrence of *Cryptosporidium hominis* in children attending day-care centers in Medellin, Colombia. *Parasitol Res* 119, 2935-2942.

Garcia, R.J., French, N., Pita, A., Velathanthiri, N., Shrestha, R., Hayman, D. (2017) Local and global genetic diversity of protozoan parasites: Spatial distribution of *Cryptosporidium* and *Giardia* genotypes. *PLoS Negl Trop Dis* 11, e0005736.

Garcia, R.J., Pita, A.B., Velathanthiri, N., French, N.P., Hayman, D.T.S. (2020) Species and genotypes causing human cryptosporidiosis in New Zealand. *Parasitol Res* 119, 2317-2326.

Ghafari, R., Rafiei, A., Tavalla, M., Moradi Choghakabodi, P., Nashibi, R., Rafiei, R. (2018) Prevalence of *Cryptosporidium* species isolated from HIV/AIDS patients in southwest of Iran. *Comp Immunol Microbiol Infect Dis* 56, 39-44.

Ghaffari, S., Kalantari, N. (2014) A multi-locus study of *Cryptosporidium* parasites isolated from patients living in Iran, Malawi, Nigeria, the United kingdom, and Vietnam. *Iranian J Parasitol* 9, 79-89.

Guy, R.A., Yanta, C.A., Muchaal, P.K., Rankin, M.A., Thivierge, K., Lau, R., Boggild, A.K. (2021) Molecular characterization of *Cryptosporidium* isolates from humans in Ontario, Canada. *Parasit Vectors* 14, 69.

Hatalova, E., Valencakova, A., Luptakova, L., Spalkova, M., Kalinova, J., Halanova, M., Bednarova, V., Gabzdilova, J., Dedinska, K., Ondriska, F., Boldis, V. (2018) The first report of animal genotypes of *Cryptosporidium parvum* in immunosuppressed and immunocompetent humans in Slovakia. *Transbound Emerg Dis* 66, 243-249.

Higuera, A., Villamizar, X., Herrera, G., Giraldo, J.C., Vasquez, A.L., Urbano, P., Villalobos, O., Tovar, C., Ramirez, J.D. (2020) Molecular detection and genotyping of intestinal protozoa from different biogeographical regions of Colombia. *PeerJ* 8, e8554.

Hijjawi, N., Mukbel, R., Yang, R., Ryan, U. (2016) Genetic characterization of *Cryptosporidium* in animal and human isolates from Jordan. *Vet Parasitol* 228, 116-120.

Hijjawi, N., Ng, J., Yang, R., Atoum, M.F., Ryan, U. (2010) Identification of rare and novel *Cryptosporidium* GP60 subtypes in human isolates from Jordan. *Exp Parasitol* 125, 161-164.

Hijjawi, N., Zahedi, A., Kazaleh, M., Ryan, U. (2017) Prevalence of *Cryptosporidium* species and subtypes in paediatric oncology and non-oncology patients with diarrhoea in Jordan. *Infect Genet Evol* 55, 127-130.

Insulander, M., Silverlas, C., Lebbad, M., Karlsson, L., Mattsson, J.G., Svenungsson, B. (2013) Molecular epidemiology and clinical manifestations of human cryptosporidiosis in Sweden. *Epidemiol Infect* 141, 1009-1020.

Iqbal, A., Goldfarb, D.M., Slinger, R., Dixon, B.R. (2015) Prevalence and molecular characterization of *Cryptosporidium* spp. and *Giardia duodenalis* in diarrhoeic patients in the Qikiqtani Region, Nunavut, Canada. *Int J Circumpolar Health* 74, 27713.

Iqbal, A., Lim, Y.A., Surin, J., Sim, B.L. (2012) High diversity of *Cryptosporidium* subgenotypes identified in Malaysian HIV/AIDS individuals targeting gp60 gene. *PLoS ONE* 7, e31139.

Iqbal, J., Khalid, N., Hira, P.R. (2011) Cryptosporidiosis in Kuwaiti children: association of clinical characteristics with *Cryptosporidium* species and subtypes. *J Med Microbiol* 60, 647-652.

Izadi, M., Jonaidi-Jafari, N., Saburi, A., Eyni, H., Rezaiemanesh, M.R., Ranjbar, R. (2012) Prevalence, molecular characteristics and risk factors for cryptosporidiosis among Iranian immunocompromised patients. *Microbiology Immunol* 56, 836-842.

Kiani, H., Haghighi, A., Seyyedtabaei, S.J., Azargashsb, E., Zebardast, N., Taghipour, N., Rostami, A., Xiao, L. (2017) Prevalence, clinical manifestations and genotyping of *Cryptosporidium* spp. in patients with gastrointestinal illnesses in western Iran. *IranianJ Parasit* 12, 169-176.

Koehler, A.V., Bradbury, R.S., Stevens, M.A., Haydon, S.R., Jex, A.R., Gasser, R.B. (2013) Genetic characterization of selected parasites from people with histories of gastrointestinal disorders using a mutation scanning-coupled approach. *Electrophoresis* 34, 1720-1728.

Koehler, A.V., Whipp, M., Hogg, G., Haydon, S.R., Stevens, M.A., Jex, A.R., Gasser, R.B. (2014) First genetic analysis of *Cryptosporidium* from humans from Tasmania, and identification of a new genotype from a traveller to Bali. *Electrophoresis* 35, 2600-2607.

Kurniawan, A., Dwintasari, S.W., Connelly, L., Nichols, R.A., Yunihastuti, E., Karyadi, T., Djauzi, S. (2013) *Cryptosporidium* species from human immunodeficiency-infected patients with chronic diarrhea in Jakarta, Indonesia. *Ann Epidemiol* 23, 720-723.

Lebbad, M., Winiecka-Krusnell, J., Stensvold, C.R., Beser, J. (2021) High diversity of *Cryptosporidium* species and subtypes identified in cryptosporidiosis acquired in Sweden and abroad. *Pathogens* 10.

Lim, Y.A., Iqbal, A., Surin, J., Sim, B.L., Jex, A.R., Nolan, M.J., Smith, H.V., Gasser, R.B. (2011) First genetic classification of *Cryptosporidium* and *Giardia* from HIV/AIDS patients in Malaysia. *Infect Genet Evol* 11, 968-974.

Loeck, B.K., Pedati, C., Iwen, P.C., McCutchen, E., Roellig, D.M., Hlavsa, M.C., Fullerton, K., Safranek, T., Carlson, A.V. (2020) Genotyping and subtyping *Cryptosporidium* to identify risk factors and transmission patterns - Nebraska, 2015-2017. *Morb Mortal Wkly Rep* 69, 335-338.

Ma, D.W., Lee, M.R., Hong, S.H., Cho, S.H., Lee, S.E. (2019) Molecular prevalence and genotypes of *Cryptosporidium parvum* and *Giardia duodenalis* in patients with acute diarrhea in Korea, 2013-2016. *Korean J Parasitol* 57, 531-536.

Mahdavi Poor, B., Rashedi, J., Asgharzadeh, M., Fallah, E., Hatam-Nahavandi, K., Dalimi, A. (2015) Molecular Characterization of *Cryptosporidium* Species in Children with Diarrhea in North West of Iran. *Int J Mol Cellular Med* 4, 235-239.

Martin-Ampudia, M., Mariscal, A., Lopez-Gigosos, R.M., Mora, L., Fernandez-Crehuet, J. (2012) Under-notification of cryptosporidiosis by routine clinical and laboratory practices among non-hospitalised children with acute diarrhoea in Southern Spain. *Infection* 40, 113-119.

Mergen, K., Espina, N., Teal, A., Madison-Antenucci, S. (2020) Detecting *Cryptosporidium* in stool samples submitted to a reference laboratory. *Am J Trop Med Hyg* 103, 421-427.

Navarro, I.M.L., da Silva, A.J., Llovo Taboada, J., Del Aguila, C., Pieniazek, N.J., Bornay-Llinares, F.J. (2013) [Molecular characterization of *Cryptosporidium* spp. isolated in humans in two different locations in Spain]. *Enferm Infecc Microbiol Clin* 31, 506-510.

Nazemalhosseini-Mojarad, E., Haghighi, A., Taghipour, N., Keshavarz, A., Mohebi, S.R., Zali, M.R., Xiao, L. (2011) Subtype analysis of *Cryptosporidium parvum* and *Cryptosporidium hominis* isolates from humans and cattle in Iran. *Vet Parasitol* 179, 250-252.

Neira, O.P., Munoz, S.N., Wilson, L.G., Barthel, M.M., Rosales, L.M., Henriquez, R.C. (2012) [*Cryptosporidium* species in immunodeficient and immunocompetent patients of Valparaiso: a descriptive study]. *Rev Chilena Infectol* 29, 63-71.

Netor Velasquez, J., Marta, E., Alicia di Risio, C., Echart, C., Gancedo, E., Victor Chertcoff, A., Bruno Malandrini, J., German Astudillo, O., Carnevale, S. (2012) Molecular identification of protozoa causing AIDS-associated cholangiopathy in Buenos Aires, Argentina. *Acta Gastroenterol Latinoam* 42, 301-308.

Network, A.C.N. (2010) Laboratory-based surveillance for *Cryptosporidium* in France, 2006-2009. *Euro Surveill* 15, 19642.

Ng-Hublin, J.S.Y., Combs, B., Reid, S., Ryan, U. (2017) Differences in the occurrence and epidemiology of cryptosporidiosis in Aboriginal and non-Aboriginal people in Western Australia (2002-2012). *Infect Genet Evol* 53, 100-106.

Ng, J., MacKenzie, B., Ryan, U. (2010) Longitudinal multi-locus molecular characterisation of sporadic Australian human clinical cases of cryptosporidiosis from 2005 to 2008. *Exp Parasitol* 125, 348-356.

Nic Lochlainn, L.M., Sane, J., Schimmer, B., Mooij, S., Roelfsema, J., van Pelt, W., Kortbeek, T. (2019) Risk factors for sporadic cryptosporidiosis in the Netherlands: analysis of a 3-year population based case-control study coupled with genotyping, 2013-2016. *J Infect Dis* 219, 1121-1129.

O'Leary, J.K., Blake, L., Corcoran, D., Elwin, K., Chalmers, R., Lucey, B., Sleator, R.D. (2020a) *Cryptosporidium* spp surveillance and epidemiology in Ireland: a longitudinal cohort study employing duplex real-time PCR based speciation of clinical cases. *J Clin Pathol* 73, 758-761.

O'Leary, J.K., Blake, L., Corcoran, G.D., Sleator, R.D., Lucey, B. (2020b) Increased diversity and novel subtypes among clinical *Cryptosporidium parvum* and *Cryptosporidium hominis* isolates in Southern Ireland. *Exp Parasitol* 218, 107967.

Osman, M., El Safadi, D., Benamrouz, S., Guyot, K., Dei-Cas, E., Aliouat el, M., Creusy, C., Mallat, H., Hamze, M., Dabboussi, F., Viscogliosi, E., Certad, G. (2015) Initial data on the molecular epidemiology of cryptosporidiosis in Lebanon. *PLoS ONE* 10, e0125129.

Peralta, R.H., Velasquez, J.N., Cunha Fde, S., Pantano, M.L., Sodre, F.C., Silva, S., Astudillo, O.G., Peralta, J.M., Carnevale, S. (2016) Genetic diversity of *Cryptosporidium* identified in clinical samples from cities in Brazil and Argentina. *Mem Inst Oswaldo Cruz* 111, 30-36.

Petrincova, A., Valencakova, A., Luptakova, L., Ondriska, F., Kalinova, J., Halanova, M., Danisova, O., Jarcuska, P. (2015) Molecular characterization and first report of *Cryptosporidium* genotypes in human population in the Slovak Republic. *Electrophoresis* 36, 2925-2930.

Pollock, K.G., Ternent, H.E., Mellor, D.J., Chalmers, R.M., Smith, H.V., Ramsay, C.N., Innocent, G.T. (2010) Spatial and temporal epidemiology of sporadic human cryptosporidiosis in Scotland. *Zoonoses Public Health* 57, 487-492.

Rafiei, A., Rashno, Z., Samarbafzadeh, A., Khademvatan, S. (2014) Molecular Characterization of *Cryptosporidium* spp. isolated from immunocompromised patients and children. *Jundishapur J Microbiol* 7, e9183.

Ramo, A., Quilez, J., Vergara-Castiblanco, C., Monteagudo, L., Del Cacho, E., Clavel, A. (2015) Multilocus typing and population structure of *Cryptosporidium* from children in Zaragoza, Spain. *Infect Genet Evol* 31, 190-197.

Ranjbar, R., Baghaei, K., Nazemalhosseini Mojarad, E. (2016) Genetic characterization of *Cryptosporidium* spp. among patients with gastrointestinal complaints. *Gastroenterol Hepatol Bed Bench* 9, 301-307.

Rolando, R.F., Silva, S., Peralta, R.H., Silva, A.J., Cunha Fde, S., Bello, A.R., Peralta, J.M. (2012) Detection and differentiation of *Cryptosporidium* by real-time polymerase chain reaction in stool samples from patients in Rio de Janeiro, Brazil. *Mem Inst Oswaldo Cruz* 107, 476-479.

Sahimin, N., Douadi, B., Yvonne Lim, A.L., Behnke, J.M., Mohd Zain, S.N. (2018) Distribution of *Giardia duodenalis* (Assemblages A and B) and *Cryptosporidium parvum* amongst migrant workers in Peninsular Malaysia. *Acta Trop* 182, 178-184.

Sanchez, A., Munoz, M., Gomez, N., Tabares, J., Segura, L., Salazar, A., Restrepo, C., Ruiz, M., Reyes, P., Qian, Y., Xiao, L., Lopez, M.C., Ramirez, J.D. (2017) Molecular Epidemiology of *Giardia*, Blastocystis and *Cryptosporidium* among Indigenous Children from the Colombian Amazon Basin. *Front Microbiol* 8, 248.

Segura, R., Prim, N., Montemayor, M., Valls, M.E., Munoz, C. (2015) Predominant virulent IbA10G2 subtype of *Cryptosporidium hominis* in human isolates in Barcelona: a five-year study. *PLoS ONE* 10, e0121753.

Sharbatkhori, M., Nazemalhosseini Mojarad, E., Taghipour, N., Pagheh, A.S., Mesgarian, F. (2015) Prevalence and genetic characterization of *Cryptosporidium* spp. in diarrheic children from Gonbad Kavoos City, Iran. *Iranian J Parasitol* 10, 441-447.

Stensvold, C.R., Ethelberg, S., Hansen, L., Sahar, S., Voldstedlund, M., Kemp, M., Hartmeyer, G.N., Otte, E., Engsbro, A.L., Nielsen, H.V., Molbak, K. (2015) *Cryptosporidium* infections in Denmark, 2010-2014. *Dan Med J* 62, 5.

Taghipour, N., Nazemalhosseini-Mojarad, E., Haghighi, A., Rostami-Nejad, M., Romani, S., Keshavarz, A., Alebouyeh, M., Zali, M. (2011) Molecular epidemiology of cryptosporidiosis in Iranian children, tehran, iran. *Iranian J Parasitol* 6, 41-45.

Urrea-Quezada, A., Gonzalez-Diaz, M., Villegas-Gomez, I., Durazo, M., Hernandez, J., Xiao, L., Valenzuela, O. (2018) Clinical manifestations of cryptosporidiosis and identification of a new *Cryptosporidium* subtype in patients from Sonora, Mexico. *Pediatr Infect Dis J* 37, e136-e138.

Valenzuela, O., Gonzalez-Diaz, M., Garibay-Escobar, A., Burgara-Estrella, A., Cano, M., Durazo, M., Bernal, R.M., Hernandez, J., Xiao, L. (2014) Molecular characterization of *Cryptosporidium* spp. in children from Mexico. *PLoS ONE* 9, e96128.

Velasquez, D.E., Arvelo, W., Cama, V.A., Lopez, B., Reyes, L., Roellig, D.M., Kahn, G.D., Lindblade, K.A. (2011) Short report: Molecular insights for *Giardia*, *Cryptosporidium*, and soil-transmitted helminths from a facility-based surveillance system in Guatemala. *Am J Trop Med Hyg* 85, 1141-1143.

Vieira, P.M., Mederle, N., Lobo, M.L., Imre, K., Mederle, O., Xiao, L., Darabus, G., Matos, O. (2015) Molecular characterisation of *Cryptosporidium* (Apicomplexa) in children and cattle in Romania. *Folia Parasitol (Praha)* 62, 002.

Villamizar, X., Higuera, A., Herrera, G., Vasquez, A.L., Buitron, L., Munoz, L.M., Gonzalez, C.F., Lopez, M.C., Giraldo, J.C., Ramirez, J.D. (2019) Molecular and descriptive epidemiology of intestinal protozoan parasites of children and their pets in Cauca, Colombia: a cross-sectional study. *BMC Infect Dis* 19, 190.

Waldron, L.S., Dimeski, B., Beggs, P.J., Ferrari, B.C., Power, M.L. (2011) Molecular epidemiology, spatiotemporal analysis, and ecology of sporadic human cryptosporidiosis in Australia. *Appl Environ Microbiol* 77, 7757-7765.

Xiao, L. (2010) Molecular epidemiology of cryptosporidiosis: an update. *Exp Parasitol* 124, 80-89.

Yang, X., Guo, Y., Xiao, L., Feng, Y. (2021) Molecular epidemiology of human cryptosporidiosis in low- and middle-income countries. *Clin Microbiol Rev* 34, e00087-00019.

Zintl, A., Ezzaty-Mirashemi, M., Chalmers, R.M., Elwin, K., Mulcahy, G., Lucy, F.E., T, D.E.W. (2011) Longitudinal and spatial distribution of GP60 subtypes in human cryptosporidiosis cases in Ireland. *Epidemiol Infect*, 139, 1945-1955.
